# Antibacterial Activities of Aliphatic Polyester Nanocomposites with Silver Nanoparticles and/or Graphene Oxide Sheets

**DOI:** 10.3390/nano9081102

**Published:** 2019-08-01

**Authors:** Chengzhu Liao, Yuchao Li, Sie Chin Tjong

**Affiliations:** 1 Department of Materials Science and Engineering, Southern University of Science and Technology, Shenzhen 518055, China; 2 Department of Materials Science and Engineering, Liaocheng University, Liaocheng 252000, China; 3 Department of Physics, City University of Hong Kong, Tat Chee Avenue, Kowloon, Hong Kong 999077, China

**Keywords:** scaffold, antibacterial activity, aliphatic polyester, osteoblast, cellular viability, staphylococcus aureus, *Escherichia coli*, electrospinning, graphene oxide, silver nanoparticle

## Abstract

Aliphatic polyesters such as poly(lactic acid) (PLA), polycaprolactone (PCL) and poly(lactic-co-glycolic) acid (PLGA) copolymers have been widely used as biomaterials for tissue engineering applications including: bone fixation devices, bone scaffolds, and wound dressings in orthopedics. However, biodegradable aliphatic polyesters are prone to bacterial infections due to the lack of antibacterial moieties in their macromolecular chains. In this respect, silver nanoparticles (AgNPs), graphene oxide (GO) sheets and AgNPs-GO hybrids can be used as reinforcing nanofillers for aliphatic polyesters in forming antimicrobial nanocomposites. However, polymeric matrix materials immobilize nanofillers to a large extent so that they cannot penetrate bacterial membrane into cytoplasm as in the case of colloidal nanoparticles or nanosheets. Accordingly, loaded GO sheets of aliphatic polyester nanocomposites have lost their antibacterial functions such as nanoknife cutting, blanket wrapping and membrane phospholipid extraction. In contrast, AgNPs fillers of polyester nanocomposites can release silver ions for destroying bacterial cells. Thus, AgNPs fillers are more effective than loaded GO sheets of polyester nanocomposiites in inhibiting bacterial infections. Aliphatic polyester nanocomposites with AgNPs and AgNPs-GO fillers are effective to kill multi-drug resistant bacteria that cause medical device-related infections.

## 1. Introduction

Biocompatibility and antibacterial properties are the most important factors to take into consideration in designing novel biomaterials for fabricating medical devices and bone scaffolds. Bacterial infection of medical devices and wounds increases significant morbidity and mortality, and has posed therapeutic challenges for healthcare worldwide [1,2,3]. Biofilms can form on medical devices and wounds as a result of bacterial contamination. Biofilm formation on a biotic (tissue) or abiotic (indwelling medical device) surfaces proceeds through three stages including an initial attachment of a bacteria species, its subsequent maturation, and final detachment [1]. For a biotic surface, the first stage involves the adhesion of bacterial surface-anchored proteins, terming as microbial surface components recognizing adhesive matrix molecules (MSCRAMMS). These adhesins can bind to human matrix proteins like fibronectin, fibrogen, collagen and vitronectin. For an abiotic surface, bacteria can attach through electrostatic and hydrophobic interactions [3,4,5,6]. The initial attachment of bacteria to a surface is mediated through electrostatic attraction or repulsion. By overcoming repulsive barrier, attachment to the surface occurs through hydrophobic interactions between bacterial adhesins and the surface [5]. Upon attachment, bacterial cells then communicate with each other by means of quorum-sensing (QS). The QS system enables the cells to display a unified response and regulates the whole stages of biofilm formation [7]. The maturation phase occurs through the excretion of extracellular polymeric substance (EPS), e.g., exopolysaccharides, extracellular DNA (eDNA) and amyloid proteins [8]. In the final stage of biofilm development, clusters of bacteria cells detach from the biofilm so that they spread and colonize new surfaces. Moreover, a small subpopulation of inactive or dormant variants of bacterial cells within the film, i.e., the ‘persister’, exhibits antibiotic tolerance and can turn active when the antimicrobial agent is removed [9]. Thus, implant-related infections are serious complications for patients because they can induce a severe inflammatory response in the surrounding tissues such as osteomyelitis, endocarditis, urinary tract infection, etc. [1,10,11,12]. 

Polymers with excellent processability and moldability, low price as well as light weight have found attractive applications in biomedical sector [13,14,15,16,17,18]. In particular, biodegradable aliphatic polyesters including polylactic acid (PLA), polyglycolic acid (PGA), poly(lactic-co-glycolic) acid (PLGA) and polycaprolactone acid (PCL) have been used extensively for bone tissue scaffolds, bone screws, bone plates, bone nails and surgical sutures in orthopedics [19,20,21,22,23,24,25]. Biodegradable aliphatic polyesters decompose through ester hydrolysis processes, leading to the scission of polymeric chains into compounds of low molecular weights. Semicrystalline PLA shows a relatively high melting point of 173–178 °C, a glass transition temperature (T_g_) of 60–65 °C, and a modulus of elasticity of 2.7–3.8 GPa [23,26]. The methyl groups in PLA render it more hydrophobic with a lower degradation rate than PGA. As such, it requires several months to years to disintegrate upon implantation to human body. PGA is hydrophilic that degrades rapidly in an aqueous solution [25]. To regulate the hydrolytic degradation, different lactic acid (LA)/glycolic acid (GA) molar ratios (90/10, 80/20, 70/30, and 50/50) are polymerized to yield PLGA copolymers. PCL exhibits the lowest melting temperature (58–63 °C) and T_g_ (−60 °C), as well as smallest modulus of elasticity (≤0.4 GPa) [27,28]. 

The scaffolds for bone tissue engineering applications generally should exhibit several favorable properties including biodegradation, biocompatibility, osteoconduction, osteoinduction, osteointegration, pore interconnectivity, vascularization, and high mechanical strength [29,30]. Accordingly, bone scaffolds provide a mechanical support for osteoblastic cell adhesion, differentiation and proliferation. In addition, bone scaffolds, bone screws and bone fixation devices should also exhibit antibacterial resistance. Contamination of environmental surfaces in hospitals is a serious issue due to the transmission of nosocomial pathogens. Gram-positive and Gram-negative bacteria can form biofilms on implanted medical devices causing infections. These include *Enterococcus faecalis*, *S. aureus* (*Staphylococcus aureus*), *S. epidermidis*, *E. coli* (*Escherichia coli*), *Klebsiella pneumoniae*, *Proteus mirabilis*, and *P. aeruginosa* (*Pseudomonas aeruginosa*). Among these species, *S. aureus* and *S. epidermidis* lead to about 40–50% prosthetic heart valve infections, 50–70% catheter biofilm infections and 87% bloodstream infections [31,32]. Gram-negative bacteria cause more than 30% of hospital-acquired infections [12]. Antibiotics are currently employed to treat bacterial infections. As the biofilm bacteria are more tolerant to antibiotics, so it becomes very difficult to treat infections due to methicillin-resistant *S. aureus* (MRSA) and multidrug-resistant *P. aeruginosa* [32,33,34]. Fibrous scaffolds that mimic the structural features of extracellular matrix are considered of clinical importance. Antimicrobial fibers can be prepared by means of electrospinning and pressurized gyration techniques [35,36,37,38,39,40,41,42,43,44,45,46]. 

In recent years, chemists and materials scientists have designed and synthesized a wide variety of nanomaterials for biomedical and industrial applications [47,48,49,50,51,52,53,54,55,56,57,58,59,60]. Among these, colloidal silver nanoparticles (AgNPs) and graphene oxide (GO) have been reported to inhibit both gram-negative and gram-positive bacterial growth greatly [57,61,62,63,64]. Earlier studies have revealed the bactericidal effect of AgNPs fillers to nondegradable synthetic polymers such as polyester and polyamide (nylon) fabrics. Non-resorbable poly(ethylene terephthalate) (PET) fibers are generally used to produce polyester fabrics [65,66,67]. Very recently, Matharu et al. reviewed antibacterial activity of several nanomaterials including AgNPs, GO, graphene platelets, copper nanoparticles, zinc oxide, iron oxide and titanium dioxide, and their polymer nanocomposites against Gram negatives and Gram positives [68]. The matrix materials used to form antimicrobial nanocomposite were mostly non-degradable polymers including polyester fabric, nylon, poly(methyl methacrylate), polypropylene, polyvinyl chloride, epoxy, etc., and inorganic molybdenum disulphide, silicon dioxide, silicon nanowires and halloysite nanotubes. They also addressed antimicrobial composites with matrices based on biodegradable chitosan, cellulose and PCL. The antibacterial performance of those polymer nanocomposites was greatly dependent on the filler contents, types of nanofillers and polymers used. For instance, the incorporation of AgNPs into polyester and nylon fabrics enhanced antimicrobial properties of the textiles typically used in hospitals and hotels. Nondegradable composite fabrics with AgNPs were unsuitable to make bone scaffolds due to a need of second surgical procedure to remove them. As recognized, natural chitosan and cellulose exhibit poor mechanical strength, thus limiting their applications as bone scaffolds. Accordingly, Xu et al. prepared antimicrobial PCL-AgNPs composite fibers with diameters of micrometer scale using pressurized melt gyration process [39]. 

Two-dimensional GO sheet with a large surface area often serves as an effective site for nucleating AgNPs. In this respect, GO-AgNP nanocomposite sheets exhibit high antibacterial activity against *S. aureus*, *E. coli* [69,70], and *P. aeruginosa* [71]. The nanocomposite sheets show a 100% inhibition rate for *P. aeruginosa* adhered on stainless steel surfaces, one of the leading bacteria causing nosocomial infections [72]. The additions of those nanofillers to aliphatic polyesters have led to the development of antibacterial nanocomposites for clinical applications. This article reviews the latest development, bactericidal and mechanical properties of biodegradable aliphatic polyester nanocomposites and scaffolds reinforced with AgNPs and/or GO sheets for bone tissue engineering. 

## 2. Bactericidal Effects of Nanomaterials

### 2.1. Silver Nanoparticles

The synthesis of AgNPs is well documented in the literature [73,74,75,76], and briefly described herein. The wet chemical reduction route is widely adopted by the researchers to synthesize AgNPs. In the process, silver salt precursor (e.g., silver nitrate) is reduced by a reducing agent in the aqueous solution or organic solvent under the presence of a polymer-based capping agent or stabilizer. Sodium borohydride, ascorbic acid, glucose, hydrazine, sodium citrate, or ethylene glycol (EG) is employed as a reducing agent, while polyvinyl alcohol (PVA), polyvinylpyrrolidone (PVP), or polyethylene glycol (PEG) is used as a stabilizer [73]. However, this synthetic approach limits the use of AgNPs for biomedical applications due to the organic solvent and reductant involved are mostly toxic. These toxic chemicals include *N*,*N*-dimethylformamide (DMF), dimethyl sulfoxide (DMSO), hydrazine, and sodium borohydride. In this respect, biosynthesis of AgNPs using various biomolecules derived from the plant leaves, fruits and nuts has attracted considerable attention in recent years due to its cost-effective and environmentally friendly properties. This route offers greener and safer solution for the synthesis of AgNPs [77,78,79,80]. 

#### Antibacterial Activity

**Figure 1:**
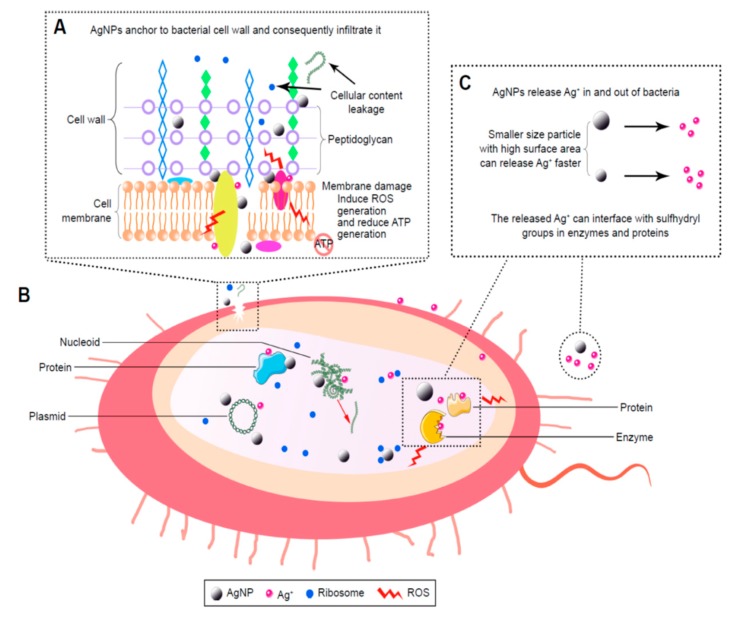
Bactericidal mechanisms of silver nanoparticles (AgNPs). (**A**) The local enlarged view shows the initial adhesion of AgNPs to the bacterial wall, leading to membrane destruction and cellular content leakage. AgNPs or silver ion (Ag^+^) can bind to the protein of cell membrane, thus inducing reactive oxygen species (ROS) generation and reducing adenosine triphosphate (ATP) production. (**B**) Penetration of AgNPs into cytoplasm; AgNPs and the released Ag^+^ can interact with proteins, enzymes, lipids, and deoxyribonucleic acid (DNA). The increased ROS levels lead to an apoptosis-like response, lipid peroxidation, and DNA damage. (**C**) AgNPs can sustainably release Ag^+^ inside and outside bacterial membrane, so Ag^+^ can interact with the proteins and enzymes accordingly.

The bactericidal effects of AgNPs have been studied extensively by many researchers, however, antimicrobial mechanisms remain unclear and subject to debate. The controversy arises from whether a direct contact of AgNPs or released silver ion from AgNPs causing bactericidal effect [54,63,73]. For a direct contact-killing mechanism [81,82,83], AgNPs in the form of metallic silver adhere to the bacterial cell wall which causes a loss in membrane integrity, leading to the leakage of cytoplasmic contents and the generation of reactive oxygen species (ROS) such as hydrogen peroxide (H_2_O_2_), hydroxyl ion (OH^−^) and superoxide anion (O_2_^−^) (Figure 1A) [64]. As is known, bacterial membranes exhibit a negative surface charge due to the presence of carboxyl and phosphate groups. There exist favorable electrostatic interactions between positively charged AgNPs and negatively charge cell membranes, thereby facilitating AgNPs attachment onto bacterial cell walls [84,85]. Thus, the adherence of AgNPs to the bacterial cell wall can be enhanced by modulating the surface charge of nanoparticles. Once attached, AgNPs can also penetrate the membrane and inactivate intracellular components such as ribosome and mitochondria, thereby inhibiting protein synthesis, causing dysfunction of respiratory enzyme system, and reducing adenosine triphosphate (ATP) production. In addition, AgNPs may interact with intracellular proteins and deoxyribonucleic acid (DNA) biomolecules, resulting in the inhibition of cell replication and the generation of ROS (Figure 1B). Morones et al. have reported that the surface interactions of AgNPs with *E. coli* reduce the permeability and respiration functions of bacterial membrane. Furthermore, AgNPs also penetrate into cytoplasm and interact with protein and DNA, leading to final cell death. Further, AgNPs can release silver ions upon residing in the cytoplasm [86]. For silver-ion mediated cytotoxicity, released silver ions tend to interact with the thiol (sulfhydryl) groups of cell wall-bound enzymes and proteins, interfering with the respiratory chain and generating ROS. Moreover, those ions can infiltrate into the cytoplasm and react with the thiol groups of cytoplasmic proteins (Figure 1C) [86]. 

**Figure 2:**
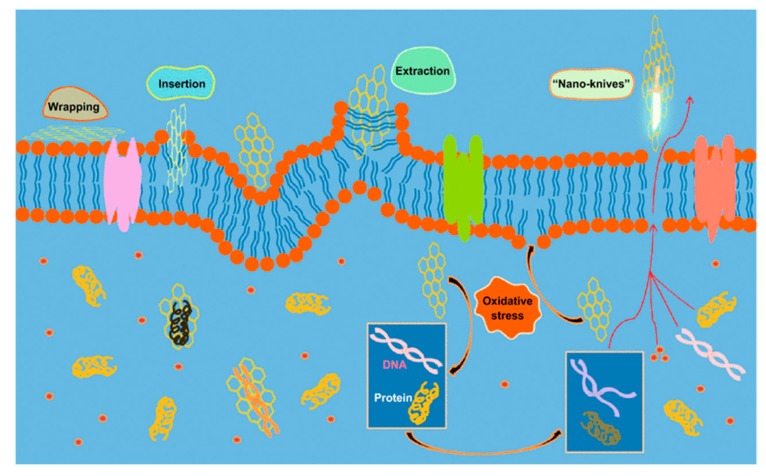
Mechanisms of the antimicrobial activities of graphene-based materials. Reprinted with permission from [57]. Copyright American Chemical Society, 2016.

In general, metals can be chemically oxidized in aerobic aqueous solutions to yield metallic ions [87,88]. Metallic nanoparticles of surface-to-volume ratio generate more ions than their micrometer scale bulk metals. Therefore, the amounts of released silver ions depend greatly on the size of AgNPs and aerobic conditions [89]. Randall et al. reported that Ag^+^ ions exhibit efficient antibacterial activities against biofilm cultures of *S. aureus* and MRSA by damaging their cell membranes [90]. Avnir and coworkers demonstrated that the entrapment of chlorhexidine (CH) within metallic silver (CH@Ag) results in the formation of a composite material with synergetic bactericidal action against *P. aeruginosa* and *S. epidermidis* [91]. The antibacterial effect derived from the co-release of Ag^+^ ions and CH from the composite. Hettinger and coworkers indicated that silver containing layers on the implant surfaces impede the growth of *S. aureus* and *E. coli* due to the release of silver ions from the coating layers [92,93]. Agnihotri et al. demonstrated that AgNPs (5 nm) exhibit the best bactericidal activity against *S. aureus* and *E. coli* than AgNPs with sizes ≥ 10 nm due to their higher released Ag^+^ levels [94]. In general, antibacterial efficacy of AgNPs is size-, shape-, dose-, and time-dependent [94,95,96,97,98]. For instance, truncated triangular silver nanoplates exhibit better bactericidal effect than spherical and rod-shaped AgNPs, due to the high silver reactivity at {111} basal plane of silver nanoplates [95]. The bactericidal efficacy of AgNPs also depends on the bacterial strains involved [84,87,97,98,99]. AgNPs have higher antibacterial activity against Gram-negative bacteria than Gram-positives owing to the difference in the structures of their cell walls. The bacterial cell wall generally locates in the outermost layer of cytoplasmic membrane, having a mesh-like network of peptidoglycan (Figure 2). The cell wall of Gram-negative bacteria is rather thin (< 10 nm), consisting of a single peptidoglycan layer, rendering easy infiltration of Ag^+^ ions to the membrane. In contrast, Gram-positive bacteria possesses a thicker (20–80 nm) peptidoglycan layer than Gram-negatives, acting as a barrier for the penetration of Ag^+^ ions into the cytoplasm [100,101]. 

### 2.2. Graphene Oxide

Graphene oxide (GO) is typically synthesized from the modified Hummers process by reacting graphite precursor and sodium nitrate with sulfuric acid and potassium permanganate [102]. As such, GO is decorated with oxygen functional groups such as carboxyl, hydroxyl and carbonyl on its basal plane and the plane edges. The presence of oxygenated functional groups reduces the elastic modulus of graphene from about 1 TPa to 207.6 ± 23.4 GPa [103], and renders it with electrical insulation behavior. Insulating GO can be chemically converted to conducting reduced graphene oxide (RGO) by adding hydrazine, sodium borohydride or hydroquinone. Hydrazine agent is commonly used for reducing GO since it can produce RGO with a relatively low oxygen content. The RGO exhibits some crystalline defects when compared with pristine graphene. Nevertheless, RGO finds attractive applications in electronics, optoelectronics, composite materials and energy-storage devices [104]. It is also commonly used as a filler material for polymers to form functional polymer nanocomposites and bionanocomposites [47,105,106,107,108,109]. The disadvantage of this chemical reduction strategy is the use of highly toxic hydrazine and sodium borohydride. In this regard, some attempts have been made by the researchers to reduce GO with eco-friendly extracts from biological plants [110,111]. Alternatively, thermal reduction route can be used to convert GO to thermally reduced graphene (TRG) through rapid heating at 1050 °C under an inert gas atmosphere [112]. 

Graphene and its derivatives are known to promote the attachment and proliferation of osteoblastic cells as well as osteogenic differentiation of bone marrow stem cells [113,114,115,116]. Accordingly, GO and RGO have been added to aliphatic polyesters to enhance bone cell adhesion and growth [117,118,119,120]. In addition, GO and RGO dispersions also exhibit good antibacterial activities [57,61,62,121,122,123,124,125,126,127,128,129,130,131]. Liu et al. exposed GO and RGO dispersions to *E. coli*, and reported that GO dispersion exhibits higher antibacterial activity than RGO [123]. GO contains abundant hydrophilic oxygen groups, while RGO is more hydrophobic due to a deficiency in oxygenated groups. Several mechanisms have been proposed to account for the bactericidal effects of graphene-based nanomaterials, including nano-knives cutting effect, membrane phospholipids extraction, oxidative stress induction, and bacterial wrapping by flexible graphene sheet as shown in Figure 2 [57,61,121,122,123,124,125,126,127,128]. Akhavan and Ghaderi reported that both GO and RGO can kill gram-positive and gram-negative bacterial strains upon direct contact with their cell envelope including cell wall and cell membrane. The direct-contact interactions begin with the insertion of ‘sharp edges’ of graphene sheet into bacterial membrane. The sharp edges of GO/RGO sheet cut and pierce through bacterial membrane, leading to the leakage of intracellular components and eventual cell death [121]. Akhavan et al. reported that flexible graphene sheet wraps *E. coli* as a blanket so that the bacteria are biologically isolated from their environment. By removing graphene sheet from the surface of microorganisms via sonication, *E. coli* bacteria reactivate and proliferate accordingly [122]. The wrapping bacteria effect is dependent on the lateral sizes of graphene sheets. Large GO sheet wraps *E. coli* effectively as a blanket and prohibits cell proliferation by limiting the supply of nutrients from the environment. This causes a large reduction in cell viability as expected. Small GO sheet exhibits a weaker bactericidal effect because of its inefficiency to isolate *E. coli* from the environment [123]. Tu et al. demonstrated that GO can extract large amounts of membrane phospholipids due to the strong van der Waals interactions between the graphene and lipid molecules, resulting in a loss of membrane integrity [124]. When GO comes in direct, physical contact with bacterial wall, it also induces oxidative stress that can give rise to phospholipid peroxidation and ROS generation, DNA damage and mitochondrial dysfunction [125,126,127,128]. Gurunathan et al. reported that the exposure of *P. aeruginosa* to GO and RGO induces significant ROS levels, leading to DNA fragmentation and final cell death. The antibacterial activities of GO and RGO are concentration- and time-dependent [127]. Very recently, Lu et al. demonstrated that the destruction process of bacterial cells by GO initiates from the cell wall envelope. The direct contact of GO would lead to a combined physical disruption of bacterial membrane through a nanoknife effect, and chemical oxidation of glutathione via a direct electron transfer mechanism for producing hydrogen peroxide [125]. They also explored the orientation or alignment of GO in damaging bacterial membrane. Glutathione is a thiol-containing peptide that is commonly used as an oxidative stress indicator. So, the oxidative stress-induced antibacterial activity relies on the electron-transfer oxidation pathway. The GO with vertical orientation is more effective in nanoknife cutting than that with horizontal or random alignment. Vertical GO contains more exposed edges to interact with bacterial glutathione, producing more oxidative stress accordingly. 

### 2.3. GO-AgNPs Nanocomposites

**Figure 3:**
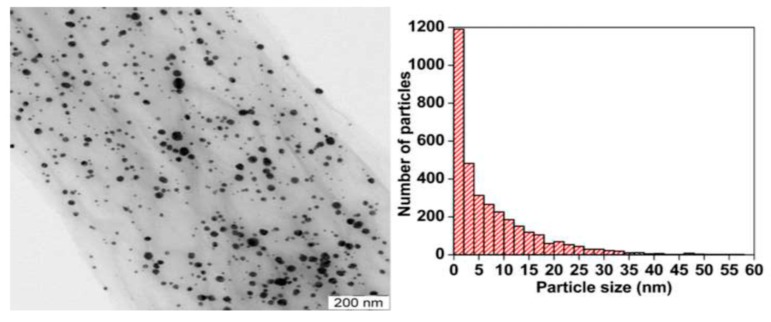
Transmission electron micrograph of GO-AgNPs (left panel) and the size distribution of AgNPs (right panel). The average size of AgNPs is 7.5 nm. Reproduced with permission from [72]. Copyright Elsevier, 2014.

Colloidal AgNPs generally tend to agglomerate into clusters in the dispersions, resulting in a decrease in their bactericidal efficiency significantly. Graphene oxide with a lateral dimension of micrometer scale often serves as an effective template for anchoring AgNPs during the synthesis process. The oxygenated groups such as carboxyl, hydroxyl, or epoxide groups of GO act as the nucleation sites for AgNPs, thereby preventing AgNPs from agglomeration. Those oxygen functional groups with negative surface charges tend to attract positively charged silver ions from silver nitrate precursor. Consequently, GO is reduced to RGO to form RGO-AgNPs nanocomposites. Furthermore, AgNPs inhibit aggregation and restacking of graphene sheet. Fined AgNPs are dispersed uniformly on the GO surface compared with large AgNPs produced without GO template (Figure 3) [132,133,134]. 

**Figure 4:**
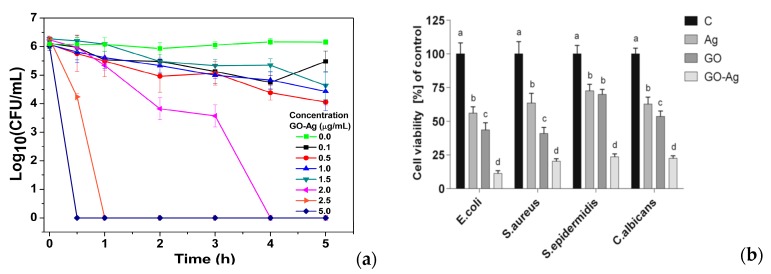

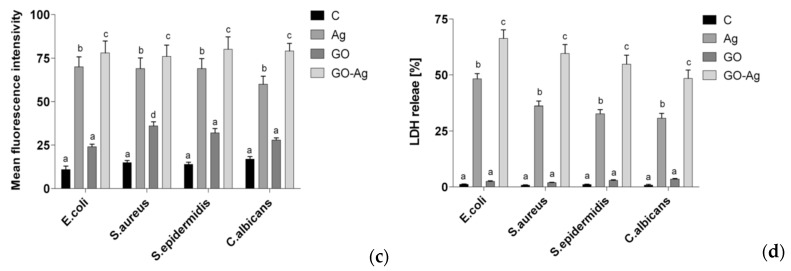
(**a**) Time-kill curves for *P. aeruginosa* at different concentrations of colloidal GO-AgNPs. The error bars represent the standard deviation of experiments performed in triplicates (*n* = 3). Reproduced with permission from [72], Copyright Elsevier, 2014. (**b**) Cell viability, (**c**) ROS level and (**d**) LDH leakage of thin foils coated with AgNPs, GO and GO-AgNPs upon exposure to *E. coli*, *S. aureus*, *S. epidermidis* and *C. albicans*; C is the control foil without nanoparticles. The columns with different letters (a–d) indicate significant differences between the foil samples exposed to bacterial strains and yeast (*p* = 0.001); error bars are standard deviations. Reproduced with permission from [137]. Copyright Springer Open, 2018.

The GO-AgNPs nanocomposites exhibit excellent antibacterial activity against several gram-negatives and gram-positives [134,135,136,137,138]. Colloidal GO-AgNPs are capable of killing *P. aeruginosa* by releasing Ag^+^ ions from finely dispersed AgNPs. Their bactericidal activity is dose- and time-dependent (Figure 4a). A marked reduction in bacterial growth is observed at GO-AgNPs contents of 2.5 and 5.0 µg/mL for 30–60 min [134]. The GO-AgNPs nanocomposites can generate higher ROS level than AgNPs or GO alone upon exposure to *S. pneumonia* and *Shigella flexneri* strains [136]. Jaworski et al. examined antibacterial efficacy of thin foils coated with GO-AgNPs, GO and AgNPs against *E. coli*, *S. aureus*, *S. epidermidis* and *C. albicans* (yeast), respectively at 37 °C for 24 h [137]. The foil with GO-AgNPs exhibited higher toxicity against those bacterial strains and yeast than the foils coated with AgNPs or GO alone. As such, GO-AgNPs foil led to a very high loss in cell viability, when compared to the foils with AgNPs or GO (Figure 4b). The amount of LDH activity acted as an indicator of membrane integrity. So damaged bacterial membranes released intracellular LDH molecules into the culture medium. Apparently, AgNPs of GO-AgNPs foil released the highest silver ions for killing bacteria due to their small sizes. Moreover, the ROS level and lactate dehydrogenase (LDH) leakage of the foil coated with GO were much lower than those of the foils with AgNPs and GO-AgNPs (Figure 4c,d). Gram-positive *S. aureus* and *S. epidermidis* with a thicker peptidoglycan layer were more resistant to the killing effect of GO-AgNPs [137]. From the literature, *S. epidermidis* bacteria often adhere to the surfaces of indwelling medical devices, hereby causing infections in prosthetic heart valves, prosthetic bones/joints, catheters, and large wounds [139]. 

## 3. Aliphatic Polyester Nanocomposites

As aforementioned, hospital acquired infections pose a significant threat to global public health and cause serious economic burden to the patients. In particular, Gram-negative bacteria cause more than 30% of hospital-acquired infections and predominate in hospital-acquired pneumonia [12]. Bacterial infection of bone implants is a major clinical concern leading to complex process of wound healing and eventual implant failure. As mentioned, mobile AgNPs can enter human cells and adversely affect intracellular functions, resulting in cytotoxicity. In this regard, some attempts have been made to incorporate AgNPs into metallic scaffolds, metallic implants, and polymeric bone grafts for combating bacterial infections [140,141,142,143,144]. Linear aliphatic polyesters that are widely employed as the materials for making bone fixation devices that are vulnerable to microbial infections. Thus, it is necessary to add AgNPs and/or GOs to aliphatic polyesters to improve their resistance to bacterial infections. Biodegradable polymer nanocomposites for bone applications can be prepared in two forms, i.e., dense film/membrane, and scaffold. In the case of dense composites, wet solvent casting and melt compounding are commonly used by the researchers. For solvent casting, polymer pellets are dissolved in a selected solvent followed by mixing with AgNPs and/or GOs fillers under sonication. The nanofiller-polymer dispersion is subsequently cast to evaporate solvent for forming nanocomposites. Melt compounding route consists of direct mixing of polymer granules/powders and nanofillers in a laboratory small-scale batch mixer or a large-volume extruder. The extruder generates homogeneous nanocomposite extrudates, which are subsequently cut into pellets by a pelletizer. Those pellets are loaded into an injection molder to produce dense nanocomposite of desired shapes. The melt compounding strategy can produce nanocomposite parts in large quantities and low cost. In contrast, solvent casting is used to fabricate a small number of nanocomposites but with a better dispersion of nanofillers in the polymer matrix. However, the use of organic solvents poses environmental and health issues. 

Porous aliphatic polyester composite scaffolds can be prepared by solvent casting/porogen leaching, electrospinning and pressurized gyration techniques [37,38,39,40,41,42,43,44,45,46,144,145,146,147,148,149,150]. Solvent casting/porogen leaching is a simple process by dissolving polymer in an organic solvent, mixing with nanofillers and porogen particles under sonication. The mixture is then cast into a thin membrane, and the porogens are removed by water leaching. The porosity level and pore size of resulting scaffolds are regulated by the amount and size of porogens added. The disadvantages of this technique include limited membrane thickness, the need of organic solvent for dissolving polymer and long processing time for removing porogen particles. Electrospinning is a versatile process for forming fibers with diameters in the range from a few nanometers to several micrometers. It also relies on the use of organic solvent for dissolving polymer granules. In the process, the polymer-filler dispersion is loaded into a syringe pump. A high voltage source is employed for generating electric field to charge the polymer-filler dispersion. At a critical electric potential, electrostatic force overcomes the surface tension of polymer dispersion, so the fluid forms a Taylor cone at a syringe tip, leading to a jet eruption from the cone. The charged jet stretches and then experiences a whipping motion towards a grounded collector, resulting in the formation of randomly oriented fibers through solvent evaporation. Well-aligned fibers can be either obtained by the rotating drum method or the deposition of fibers to the gap between two electrodes of a grounded collector. Electrospinning has received increasing attention recently as a promising tool for fabricating nanocomposite fibrous mats for biomedical engineering applications, e.g., bone tissue engineering scaffolds, drug delivery vehicles and wound dressings [36,146,147,148,149,150]. Moreover, electrospun polymer nanofibers, especially natural biodegradable polymer fibers (e.g., collagen, gelatin and chitosan) tend to mimic the structure and function of extracellular matrix (ECM), having a protein fibrous network that gives structural support to surrounding cells [150]. To avoid the use of toxic solvents, melt-electrospinning has emerged as a tool to form PCL fibers with diameters of several to hundreds of micrometers [146]. 

**Figure 5:**
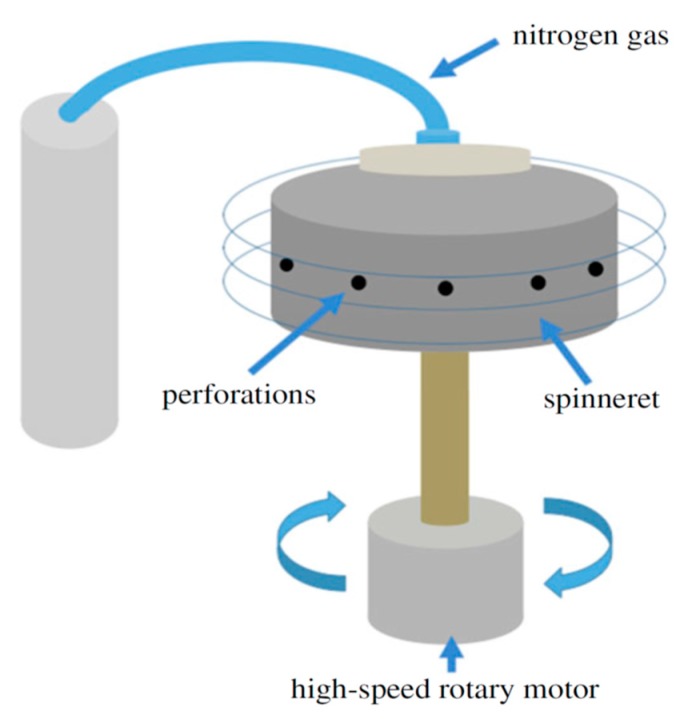
Schematic diagram of the pressurized gyration system for fabricating polymer fibers. Reproduced with permission from [44]. Copyright Royal Society, 2018.

Alternatively, pressurized gyration process can be used to generate large quantities of polymer fibers with diameters ranging from nanometer to micrometer scale [36,37,38,39,40,41,42,43,44,45,46]. This technique utilizes a centrifugal force rather than an electric field for spinning fibers, and offers advantages of high production yield, ease of fabrication and the manipulation of fiber morphology [37]. It involves rotating a perforated aluminum cylindrical vessel containing a polymer solution under a high speed and at a high pressure (Figure 5). The polymer solution emerges through the orifices, thereby generating a multitude of jets. Therefore, a combination of centrifugal spinning and solution blowing acts against the solution surface tension; resulting in the formation of fibers [36]. In one case, a syringe pump is connected to the bottom of the vessel for regulating the polymer solution flow to form nanofibers. Accordingly, the rotating speed of the vessel, air pressure, polymer solution concentration and flow rate affect the fiber diameters and morphologies greatly [41]. To avoid the use of organic solvents, Xu et al. employed pressurized melt gyration to fabricate PCL fibers. In the process, a heating gun was employed to melt PCL pellets such that the melt temperature, gyration speed and working pressure affected the fiber diameters [39]. Comparing to solution pressurized gyration, pressurized melt gyration gave rise to PCL fibers with diameters of several tens of micrometers. 

### 3.1. PLA-Based Nanocomposites

PLA is a chiral polymer with two enantiomers, l- and d-lactic acid, thereby giving rise to PLLA, PDLA and PDLLA with varied physical and biodegradation properties. In general, PLLA is more often employed for biomedical applications than PDLA. Biodegradable PLA has been widely used for making bone scaffolds and internal fixation devices in orthopedics [151]. However, PLA is susceptible to bacterial colonization due to the lack of antimicrobial moieties. Thus, AgNPs and/or GOs additions are beneficial for PLA in forming antimicrobial nanocomposites. However, PLA matrix of those nanocomposites can in turn affect antibacterial activities of AgNPs and GOs. The PLA matrix provides a surface for immobilizing AgNPs and GOs fillers, preventing them from penetrating bacterial membrane. In other words, AgNPs and GOs cannot penetrate into bacterial cytoplasm, and interact with mitochondria, ribosome, and biomolecules such as protein and DNA. Furthermore, PLA matrix would seal the sharp edges of embedded GOs and rGOs, thereby reducing their nanoknife cutting effect. As the GOs or RGOs are embedded in the PLA matrix, the wrapping bacteria effect through flexible graphene sheets is reduced/diminished accordingly. 

#### 3.1.1. PLA/AgNPs Nanocomposites

Several researchers prepared antimicrobial PLA/AgNPs nanocomposites using solvent casting and melt compounding techniques [152,153,154]. Shameli et al. fabricated solvent-cast PLA/AgNPs nanocomposites containing 8, 16 and 32 wt% AgNPs [152]. They reported that PLA/AgNPs nanocomposite films exhibited a strong antibacterial activity against Gram-negative *E. coli* and *Vibrio parahaemolyticus*, and gram-positive *S. aureus*. The antibacterial efficacy of PLA/AgNPs films towards those bacterial strains increased with increasing AgNPs content. As the AgNPs were immobilized by the PLA matrix, so their penetration into cytoplasm was restricted. The bactericidal effect of those nanocomposite films was attributed to the silver ions released from AgNPs. The amount of released silver ions from PLA/AgNPs nanocomposite films in the phosphate-buffered saline (PBS) solution was found to increase with increasing AgNPs content. 

**Figure 6:**
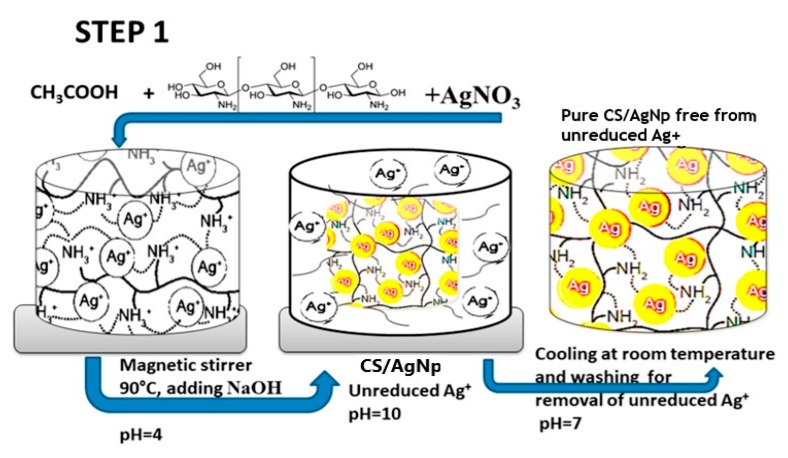

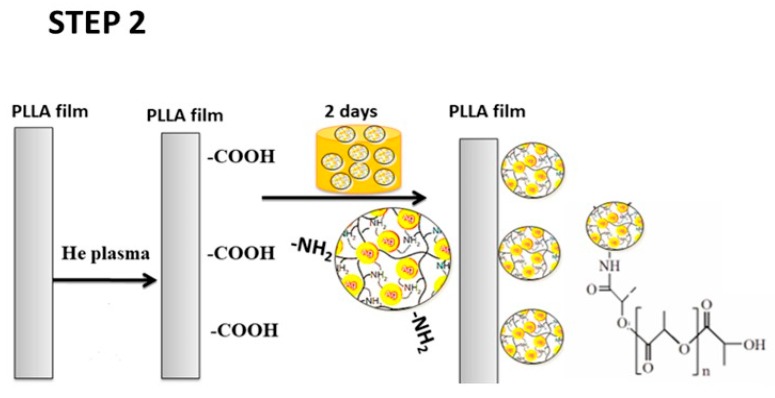
Schematic for the preparation strategy of PLA/AgNPs nanocomposite film.

**Figure 7:**
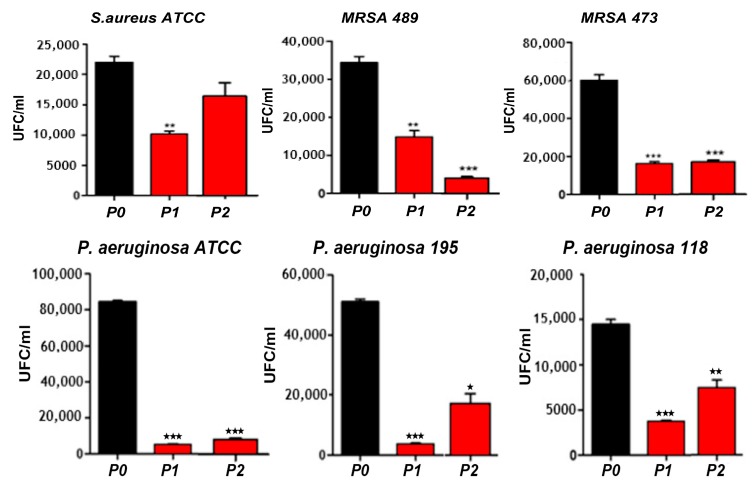
Bacterial population in colony forming unit (UFC/mL) of different *S. aureus* strains and *P. aeruginosa* strains adhered on P0, PI and P2 films. *, ** and *** correspond to the magnitude of antimicrobial activity. MRSA: Methicillin-resistant Staphylococcus aureus, and ATCC: American Type Culture Collection.

Very recently, Aflori et al. reacted chitosan (CS) with AgNO_3_ precursor to yield CS-AgNPs. CS acted as both capping and reducing agent for forming AgNPs (Figure 6; step 1). They then treated PLLA with helium plasma at 30 W for 4 min and 10 min, respectively in order to induce carboxyl groups on its surface [153]. The plasma-treated PLLA was immediately immersed in the CS-AgNPs solution at room temperature for 2 days. As such, CS-AgNPs were grafted onto PLLA due to the interactions between the carboxyl groups of plasma-treated PLLA and the amino groups of CS (Figure 6; step 2). Neat PLLA (P0), plasma-treated PLLA (4 min)/AgNPs (P1), and plasma-treated PLLA (10 min)/AgNPs (P2) films were exposed to different *S. aureus* strains and *P. aeruginosa* strains (Figure 7). *P. aeruginosa* is a leading cause of ventilator-associated pneumonia, bacteremia, and surgical wound infections [33]. Furthermore, multi-drug resistant strains of *S. aureus* and *P. aeruginosa* are often difficult to cure with conventional antibiotics. Comparing with PLLA, P1 and P2 films exhibited excellent antibacterial activity. In particular, antibacterial efficacy of P1 film was more pronounced against gram-negative *P. aeruginosa* than against *S. aureus*. This was due to *P. aeruginosa* had a thinner peptidoglycan wall. 

**Figure 8:**
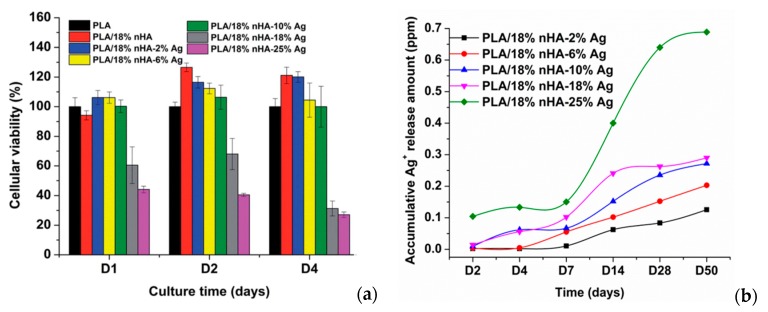
(**a**) Viability of osteoblasts cultured on PLA and its nanocomposites for 1, 2 and 4 days; error bars denoted standard deviations. (**b**) Released Ag^+^ ion vs. time plots of PLA/18 wt% nHA–AgNPs hybrids immersed in distilled water at 37 °C. The concentration of silver ions was determined with inductively coupled plasma atomic emission spectrometry. Reproduced with permission from [154], Copyright Royal Society of Chemistry, 2015.

Generally, PLA also lacks of bioactive function and osteointegration. These can be improved by adding hydroxyapatite nanorod (nHA), a major mineral constituent of human bone, for enhancing bone cell adhesion and growth. Tjong and coworkers have fabricated PLA/18 wt% nHA–(2–25 wt%)AgNPs nanocomposites using melt-mixing and injection molding [154]. Figure 8a shows the 3-(4,5-dimethylthiazol-2-yl)-2,5-diphenyltetrazolium bromide (MTT) test results for human osteoblast cell line (Saos-2) cultured on PLA/nHA-AgNPs nanocomposites for different time points. The addition of 18 wt% nHA to PLA enhances osteoblastic cell viability at day 2 and day 4. The incorporation of 2 wt% and 10 wt% AgNPs into PLA/18 wt% nHA nanocomposite leads to a slight decrease in the bone cell viability. However, osteoblastic viability reduces significantly by adding 18 wt% AgNPs and 25 wt% AgNPs to PLA/18 wt% nHA at day 2 and day 4. A marked reduction in the cell viability at high AgNPs contents can be attributed to the cytotoxic effect of released Ag^+^ ions. As AgNPs fillers are firmly bound to the PLA matrix, they cannot penetrate osteoblastic membrane, so those fillers release Ag^+^ ions in reducing the viability of Saos-2. Figure 8b shows the released Ag^+^ profiles of PLA/18 wt% nHA–AgNPs hybrids immersed in distilled water at 37 °C. The amount of Ag^+^ cation is dependent on the AgNPs loadings. For the hybrid composites with low AgNPs loadings (e.g., 2 wt%), Ag^+^ cation release rate is very slow at an earlier stage of immersion (day 2–day 7). The polymer matrix shields AgNPs from direct contact with water, thus causing slow cation release. However, Ag^+^ cation contents increase moderately at day14 and above due to the PLA degradation as a result of polymer chain scission. In contrast, larger amounts of Ag^+^ ions are released from hybrid nanocomposites with AgNPs loadings ≥ 10 wt% at day 14 and above. 

**Figure 9:**
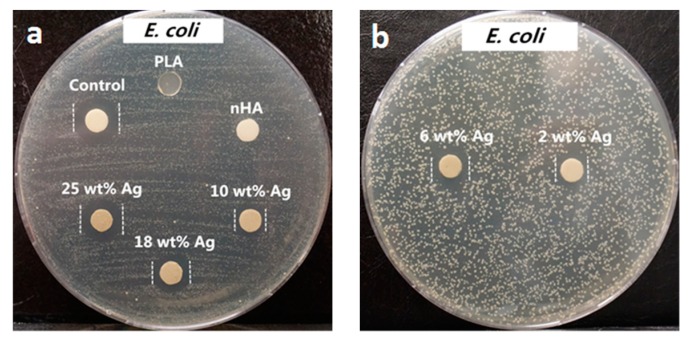
Macrographs of agar plates showing bacterial inhibition zones against *E. coli*: (**a**) control (AgNO_3_), PLA, PLA/18 wt% nHA, PLA/18 wt% nHA–10 wt% AgNPs, PLA/18 wt% nHA–18 wt% AgNPs and PLA/18 wt% nHA–25 wt% AgNPs samples, and (**b**) PLA/18 wt% nHA–2 wt% AgNPs and PLA/18 wt% nHA–6 wt% AgNPs hybrid nanocomposites. Reproduced from [154], Copyright Royal Society of Chemistry, 2015.

**Figure 10:**
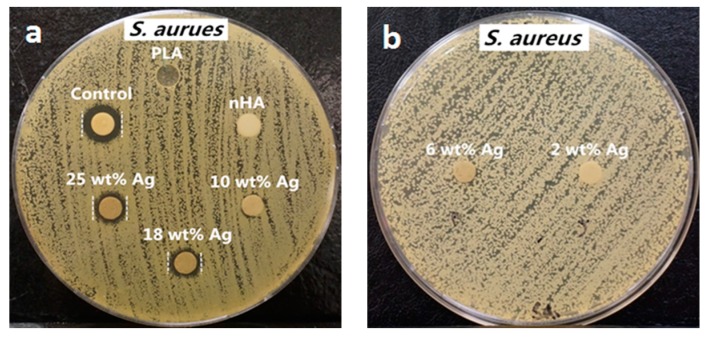
Macrographs of agar plates showing bacterial inhibition zones against *S. aureus*: (**a**) control (AgNO_3_), PLA, PLA/18 wt% nHA, PLA/18 wt% nHA–10 wt% AgNPs, PLA/18 wt% nHA–18 wt% AgNPs and PLA/18 wt% nHA–25 wt% AgNPs samples, and (**b**) PLA/18 wt% nHA–2 wt% AgNPs and PLA/18 wt% nHA–6 wt% AgNPs hybrid nanocomposites. Reproduced with permission from [154], Copyright Royal Society of Chemistry, 2015.

From the literature, AgNPs and Ag^+^ ions can also induce cytotoxicity to osteoblasts and other mammalian cells by generating ROS in a dose dependent manner, leading to mitochondrial dysfunction, DNA damage, protein denaturation, and apoptosis [155,156]. Thus, the amounts of AgNP in the PLA/18 wt% nHA–AgNPs hybrids must be regulated to avoid cytotoxic effect on osteoblasts. Figure 9a,b and Figure 10a,b show the disk diffusion test results of PLA and its hybrids upon exposure to *E. coli* and *S. aureus*, respectively. Neat PLA and PLA/18 wt% nHA exhibit no inhibition zone to *E. coli* and *S. aureus* as expected. The inhibition zone of PLA/18 wt% nHA–AgNPs hybrids against *E. coli* increases with increasing AgNP content. The bactericidal activity of these hybrids derives from the Ag^+^ ions. In addition, gram-positive *S. aureus* is less susceptible to AgNPs than *E. coli*. Hybrid nanocomposites with AgNPs contents ≤ 10 wt% exhibit no bacterial inhibition zones. The zones can be seen in the hybrids with high AgNPs contents, i.e., 18 wt% and 25 wt% AgNPs. 

Xu et al. electrospun PLA/AgNPs fibrous mats by dissolving PLA in mixed organic solvents (DMF and dichloromethane (DCM)) followed by adding AgNO_3_. The AgNO_3_ content used was 8 wt%, 16 wt% and 32 wt%, respectively, with respect to PLA. The as-spun PLA/AgNO_3_ fibers were then heat treated in a furnace at 80 °C for 48 h to reduce AgNO_3_ [157]. The average size of AgNPs in the composite fibers was 30 nm, being independent on the AgNO_3_ loading levels. The antibacterial efficacies of the PLA/AgNPs fibers against *S. aureus* and *E. coli* were 98.5% and 94.2%, respectively. The released silver ions were found to be responsible for bactericidal activity of the composite fibers by destructing bacterial cell membranes. 

#### 3.1.2. PLA/GO Nanocomposites

As mentioned, graphene and its derivatives promote the attachment and proliferation of bone cells as well as osteogenic differentiation of bone marrow stem cells [113,114,115,116]. Thus, the incorporation of GO into PLA enhances bone adhesion and growth on its surface [19,158,159]. However, very few information is available in the literature relating antibacterial activity of PLA/GO nanocomposites [160,161,162]. Arriagada et al. prepared PLA/GO and PLA/TRG nanocomposites using melt-mixing process [160]. They reported that additions of 3 wt% GO or 3 wt% TRG to PLA had little effect in reducing bacterial populations of *E. coli* and *S. aureus*. The addition of 5 wt% GO led to a reduction of *E. coli* and *S. aureus* populations. In contrast, the cell numbers of both bacterial strains changed slightly by adding 5 wt% TRG. This was ascribed to hydrophilicity of GO and hydrophobicity of TRG. By increasing the GO loading to 5 wt%, the water contact angle of PLA reduced substantially, thereby increasing hydrophilicity of the resulting nanocomposite, and forming an inhibition surface for bacterial adhesion and growth. As is generally known, hydrophilic uncharged surfaces showed the greatest resistance to bacterial cell attachment [4,163]. By applying an electrical stimulus to PLA/TRG, its antibacterial behavior was markedly increased. They suggested this to the generation of an electrostatic effect, i.e., electron transfer interaction from microbial membrane to conducting TRG, leading to bacterial cell death [160,164]. It is noted that the surfaces with hydrophilic behavior do not always inhibit bacterial attachment and colonization. Although many bacteria attach preferentially to hydrophobic surfaces, however, *S. epidermidis* bacteria favor polar, hydrophilic surfaces [5]. 

#### 3.1.3. PLA/GO-AgNPs Nanocomposites

**Figure 11:**
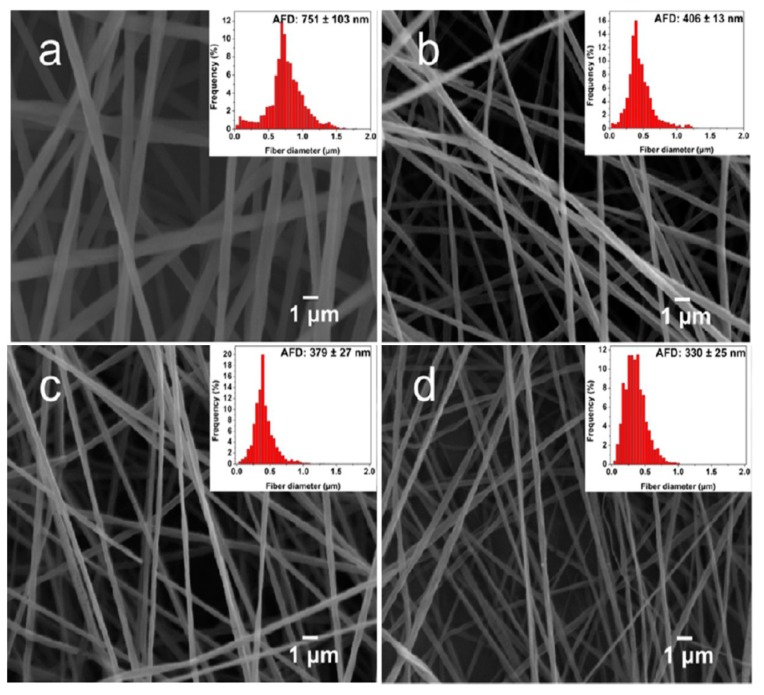
SEM images of electrospun (**a**) PLA, (**b**) PLA/1 wt% GO, (**c**) PLA/3 wt% Ag, and (**d**) PLA/1 wt% GO-3 wt% Ag fibrous mats. Reproduced from [165], Copyright American Chemical Society, 2017.

**Figure 12:**
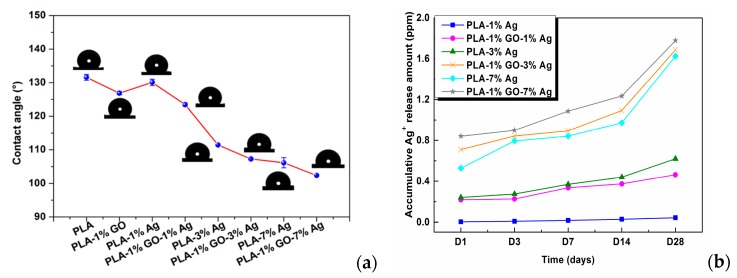
(**a**) Images of water contact angles on PLA and its nanocomposite fibrous mats, and (**b**) released Ag^+^ concentration vs. immersion time profiles of PLA/AgNPs and PLA/1 wt%GO-AgNPs mats immersed in distilled water for different time points. The concentration of silver ions was determined with inductively coupled plasma atomic emission spectrometry. Reproduced with permission from [165], Copyright American Chemical Society, 2017.

Very recently, Tjong and coworkers studied antibacterial activity of electrospun PLA/1 wt% GO-AgNPs, PLA/GO and PLA/AgNPs fibrous mats [165]. The electrospinning solutions were prepared by dissolving PLA in mixed DMF and DCM solvents followed by either adding GO, AgNPs or hybrid GO-AgNPs. Figure 11a–d show the SEM images depicting the morphologies of neat PLA, PLA/1 wt% GO, PLA/3 wt% AgNPs, and PLA/1 wt% GO–3 wt% AgNPs nanofibers. Pure PLA mat exhibits smooth and continuous fibers without forming polymer beads. PLA fibers have an average fiber diameter of 751 ± 103 nm on the basis of ImageJ software analysis (inset figure). The fiber diameters of nanocomposite fibrous mats decrease markedly by adding 1 wt% GO or AgNPs of different loading levels. By incorporating hybrid 1 wt% GO-AgNPs into PLA, fined fibers with the smallest diameters are produced. As recognized, the fiber diameter and porosity level of electrospun scaffolds can be tuned by monitoring the solution parameters including solvent type, polymer concentration, viscosity, solution conductivity, and operational factors such as the applied voltage, syringe needle diameter, and syringe needle tip to collector distance [150,166]. The additions of non-conducting fillers increase the viscosity of polymer solution, so high viscosity results in a large fiber diameter. However, conducting fillers tend to increase the solution conductivity, thereby allowing greater stretching of the fluid jet due to the presence of more charge carriers. This leads to the formation of fine composite fibers. In this respect, PLA/AgNPs and PLA/1 wt% GO-AgNPs mats exhibit smaller diameters than neat PLA mat. Figure 12a shows the water contact angle images and values for both PLA/AgNPs and PLA/1 wt% GO-AgNPs systems. The water contact angle of PLA/AgNPs system decreases with increasing AgNPs content, thereby improving the wettability of the system. The additions of AgNPs to hydrophobic polymers have been found to improve their wettability by reducing their water contact angles [167]. Similarly, GO with abundant oxygenated groups also improves the wettability of polymers. As such, PLA/AgNPs mats with high AgNPs loadings and PLA/1 wt% GO-AgNPs system exhibit less hydrophobic surfaces (Figure 12a). Therefore, PLA/7 wt% AgNPs, PLA/1 wt%GO-3 wt% AgNPs and PLA/1 wt%GO-7 wt% AgNPs fibrous mats can release a large amount of Ag^+^ ions upon immersion in distilled water (Figure 12b). It is noted that electrospun PLA fibrous mats can release more silver ions than dense PLA nanocomposite films. This is because water molecules can penetrate into fibrous mats, and degrade the PLA matrix inside the mats to expose AgNPs for releasing Ag^+^ ions. In dense nanocomposite films, water molecules can degrade only the PLA matrix of nanocomposite surfaces for exposing AgNPs. 

**Figure 13:**
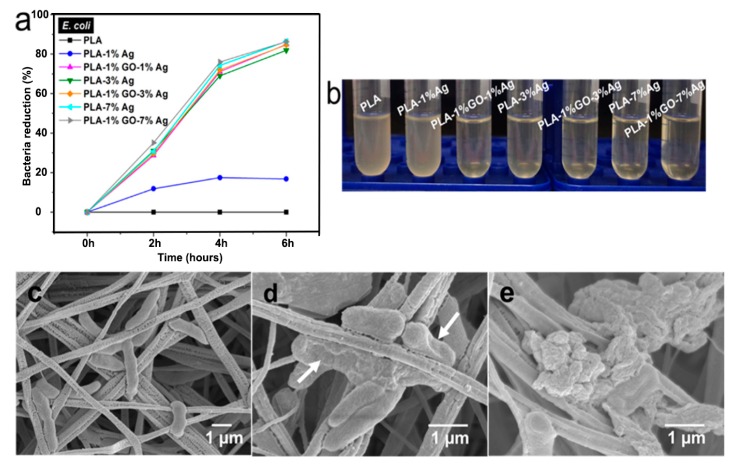
(**a**) Bacterial reduction percentage vs. time profiles for *E. coli* culture medium treated with PLA and its nanocomposite fibrous mats. The circular disk samples were soaked in the test tubes containing nutrient broth for *E. coli* (1 × 10^6^ CFU/mL), and then placed in a rotary shaker at 37 °C for different time points. (**b**) Photograph of *E. coli* culture suspensions treated with PLA and its nanocomposite fibrous mats for 6 h. SEM images showing progressive loss of membrane integrity of *E. coli* attached on PLA/1 wt%GO-3 wt%AgNPs fibers for (**c**) 2, (**d**) 4, and (**e**) 6 h. White arrows indicate ruptured cell membranes. Reproduced with permission from [165], Copyright American Chemical Society, 2017.

**Figure 14:**
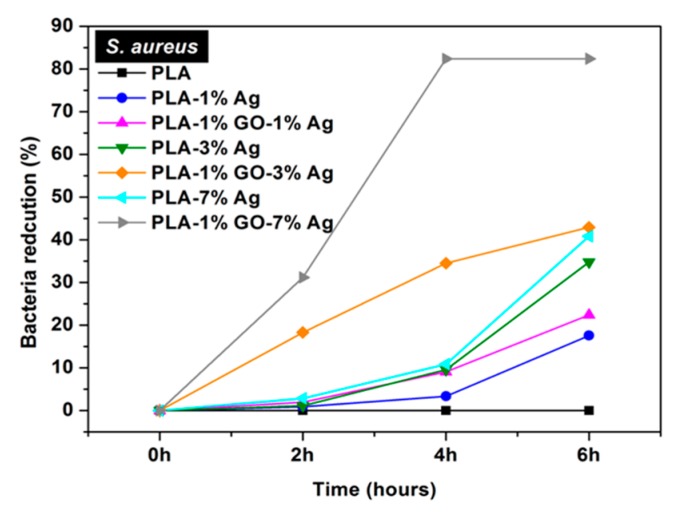
Bacterial reduction percentage vs. time profiles for *S. aureus* culture medium inoculated with electrospun PLA and its nanocomposite mats. Reproduced with permission from [165], Copyright American Chemical Society, 2017.

Figure 13a shows the antibacterial efficacy vs. time plots for electrospun PLA and its nanocomposite mats inoculated with *E. coli* culture media using a rotating shaker test at 37 °C. This turbidity test generates quantitative data by detecting the light absorbance of a bacteria culture suspension [165]. PLA mat exhibits no bactericidal activity as expected. The addition of 1 wt% AgNPs to PLA improves its bactericidal activity moderately as evidenced by a low bactericidal efficacy of about 18% after 4 h to 6 h. The antibacterial efficacy of PLA/AgNPs system increases with increasing AgNPs loading levels. The PLA/1 wt% GO-AgNPs hybrid mats also show excellent antibacterial activity owing to the Ag^+^ ions released from AgNPs fillers as shown in Figure 13b. Figure 13b reveals that the culture medium treated with PLA appears cloudy because of the ineffectiveness of PLA in killing bacteria. The culture medium with PLA/1 wt% AgNPs becomes less cloudy. In contrast, bacterial medium treated with PLA/1 wt% GO-1 wt% AgNPs for 6 h looks clear due to a high bactericidal efficacy of 83%. Clear culture dispersions are also observed after treating with PLA/AgNPs and PLA/1 wt% GO-AgNPs systems of high AgNPs loadings. Figure 13c–e are the SEM images displaying progressive membrane destruction of *E. coli* treated with PLA/1 wt% GO-3 wt% AgNPs fibers. At 4 h, the membrane integrity of *E. coli* is lost as evidenced by the rupture of some cell membranes (indicated by the arrows) leading to cell lysis and apoptosis. As a consequence, decayed cell debris are observed at 6 h. In general, gram-positive *S. aureus* with a thick peptidoglycan layer exhibit strong resistance to PLA/AgNPs system (Figure 14). The suspensions with PLA/7 wt%AgNPs and PLA/1 wt%GO-3 wt% AgNPs exhibit low antibacterial efficacy of 41% at 6 h with the exception of PLA/1 wt%GO-7 wt% AgNPs mat. This hybrid mat exhibits high antibacterial efficacy of 82% at 6 h. 

**Figure 15:**
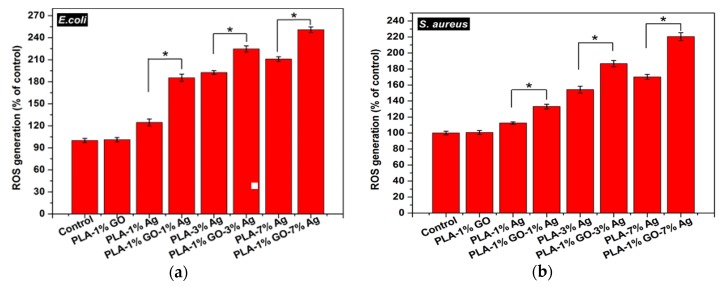
ROS levels in (**a**) *E. coli* and (**b**) *S. aureus* treated with electrospun PLA (control) and its nanocomposite mats. * denotes statistically significant difference between the test groups (* *p* < 0.05). Reprinted with permission from [165], Copyright American Chemical Society, 2017.

As described, the cell membrane disruption of *E. coli* is due to the direct contact with PLA/AgNPs and PLA/1 wt%GO-AgNPs nanocomposite fibers. As the AgNPs fillers are immobilized and embedded in the PLA matrix, it is considered that the released Ag^+^ ions from AgNPs fillers react with the thiol groups of bacteria for generating ROS [90]. The ROS generation is evaluated with the DCFH-DA assay in which 2,7-dichlorofluorescein diacetate (DCFH-DA) crosses the cell membrane. It is enzymatically cleaved by the esterase to yield nonfluorescent 2,7-dichlorofluorescein (DCFH). Further oxidation of DCFH by ROS converts the molecule to fluorescent 2,7-dichlorofluorescein (DCF) [168]. Figure 15a shows the ROS levels in *E. coli* treated with electrospun PLA and its nanocomposite fibers. Hybrid PLA/1 wt% GO-AgNPs mats induce high levels of ROS in *E. coli*. The PLA/3 wt% AgNPs and PLA/7 wt% AgNPs show slightly lower ROS levels compared with the hybrid mats. However, PLA/1 wt% GO mat exhibits low ROS level, being similar to that of PLA control. So the 1 wt% GO addition to PLA exerts no effect for bactericidal activity. In the PLA/1 wt% GO mat, the GOs are immobilized by the PLA matrix, so the polymer material seals sharp edges of graphene sheets, diminishing their role as nanoknives. Figure 15b shows that the ROS level in *S. aureus* treated with the PLA-based hybrids is also higher than that of PLA/AgNPs mat system. The ROS level in *S. aureus* treated with PLA/1 wt% GO mat is relatively low and similar to that of the PLA control. 

It is worth noting that the immobilization of GO fillers by the PLA matrix is beneficial for enhancing biocompatibility of resulting nanocomposites because the destructive effect of mobile GOs to human cells diminishes. Immobile GO fillers with a large surface area provide an effective site for osteoblastic cell adhesion and growth. GOs have been reported to promote osteogenic stem cell adhesion, differentiation and proliferation [113,114,115,116]. However, GOs can also induce a loss in human cell viability through the generation of ROS, the extraction of membrane lipids or cholesterols, and the formation of carbon radicals. GOs with small sizes can be internalized by macrophages, resulting in inflammation [116]. Thus mobile GOs act as a double-edged sword having beneficial and disadvantageous impacts on the bone cell adhesion and growth. The internalization of GOs by macrophages can be eliminated completely by adding or immobilizing GOs in the PLA matrix. 

### 3.2. PLGA-Based Nanocomposites

**Figure 16:**
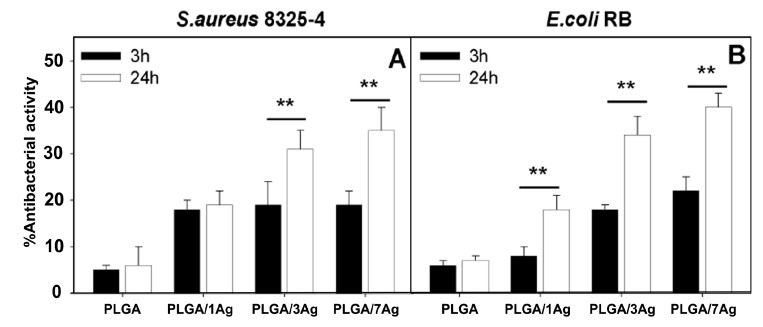
Antibacterial activity of dense PLGA and its PLGA/AgNPs nanocomposite films against (**A**) *S. aureus* and (**B**) *E. coli* incubated for 3 and 24 h at 37 °C. The percentage viability was determined by setting the bacterial cells grown onto the tissue culture plate (TCP) wells to 100%. Error bars indicated standard errors of the means, and ** denoted statistical significance, *p* < 0.01.

**Figure 17:**
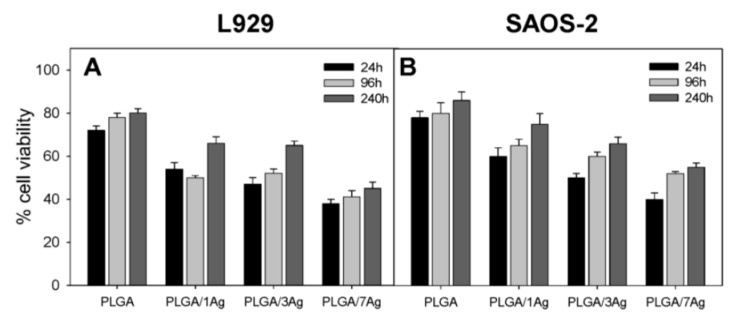
MTT test results showing cell viability of (**A**) murine fibroblasts (L929) and (**B**) human osteosarcoma cell line (Saos-2) cultured on dense PLGA and its nanocomposite films for 24 h, 96 h and 240 h. The error bars were standard deviations.

**Figure 18:**
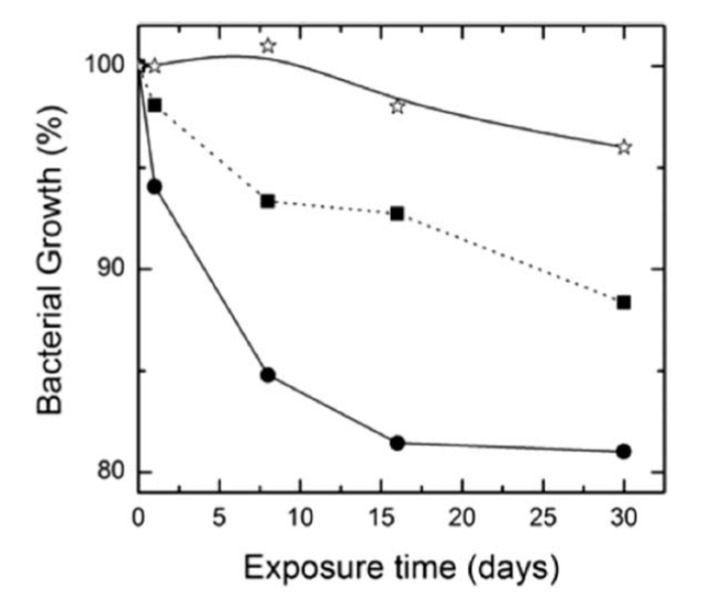
Bacterial growth vs. exposure time profiles of neat PLGA (star), PLGA/1 wt% AgNPs (square), and PLGA/3 wt% AgNPs (circle). Reproduced with permission from [170], Copyright Wiley, 2013.

The degradation rate of PGA is relatively fast in aqueous solutions, thereby limiting its biomedical applications. Therefore, PGA is usually copolymerized with PLA to produce PLGA. More recently, Scavone et al. fabricated dense PLGA/AgNPs nanocomposite films with 1 wt%, 3 wt% and 7 wt% AgNPs by means of solvent casting [169]. The antibacterial activity of solvent-cast PLGA/AgNPs system was studied (Figure 16). Apparently, dense PLGA/AgNPs films exhibited a higher antibacterial resistance against *E. coli* and *S. aureus* over neat PLGA. The antibacterial activity of dense PLGA/AgNPs films derived from the released Ag^+^ ions, and increased with increasing AgNPs loadings. Figure 17A,B showed the MTT test results for murine fibroblasts (L929) and Saos-2 cultured on neat PLGA and its nanocomposite films. PLGA/7 wt% AgNPs nanocomposite film exhibited the lowest cell viability for L929 and Saos-2 cells, demonstrating cytotoxic effect of AgNPs with a high filler loading on mammalian cells. Rinaldi et al. also fabricated solvent-cast PLGA/(1–3 wt%) AgNPs nanocomposite films and examined their bactericidal activity against *E. coli* [170]. The antibacterial activity of PLGA/(1–3 wt%) AgNPs nanocomposite films increased with silver loading (Figure 18). 

#### Porous PLGA/AgNPs Scaffolds

Zheng et al. fabricated porous PLGA/AgNPs grafts using solvent casting/porogen process. Commercial AgNPs (QSI-Nano^®^) prepared by a patented vapor condensation process from QuantumSphere, Inc. (Santa Ana, CA, USA) were used as the fillers [144]. Subsequently, bone morphogenetic protein-2 (BMP-2) was injected into porous PLGA/(1–2%)AgNPs to create BMP-2 immobilized PLGA/(1–2%)AgNPs grafts for in vivo animal model studies. BMP-2 was an effective growth factor in bone tissue engineering. The BMP-2 coupled PLGA/(1–2%)AgNPs grafts and BMP-2 coupled PLGA (control) were inoculated with vancomycin-resistant *S. aureus*, and then implanted into femoral bone defects of mice. Infected femoral defect implanted with PLGA/2% AgNPs graft healed in 12 weeks showing no sign of residual bacteria. In contrast, the bone defect with control graft failed to heal in the presence of multi-drug resistant bacteria. The BMP-2 coupled PLGA/2% AgNPs graft exhibited good antibacterial activity without showing adverse effects on BMP-2 osteoinductivity. This implied that porous BMP-2 coupled PLGA/2%AgNPs graft shows promise for use in bone regeneration of contaminated wounds owing to its capability to kill vancomycin-resistant *S. aureus*. Vancomycin is usually prescribed for the treatment of methicillin-resistant *S. aureus* (MRSA) in the clinical sector. Zheng et al. also claimed that highly purified (>99.9%) QSI-Nano^®^ silver nanoparticles (20–40 nm) exhibit better bactericidal activity than the AgNPs prepared from chemical reduction method having residual chemical impurities and wide distribution of particle sizes [144]. As such, addition of only 2% QSI-Nano^®^ to PLGA (PLGA/2%AgNPs) was capable of releasing a sufficient amount of Ag^+^ ions for killing multi-drug resistant *S. aureus*. This viewpoint agreed reasonably with the disk diffusion test for dense PLA-based nanocomposite containing chemically reduced AgNPs (2 wt%) as depicted in Figure 11b, showing its poor resistance to *S. aureus*. 

**Figure 19:**
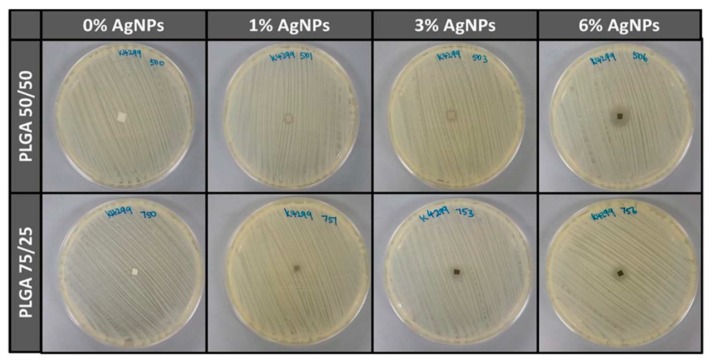
Antibacterial activity of electrospun PLGA 50/50 and PLGA 75/25 mats with and without AgNPs upon exposure to *P. aeruginosa*. Reproduced with permission from [172], Copyright Wiley, 2015.

**Figure 20:**
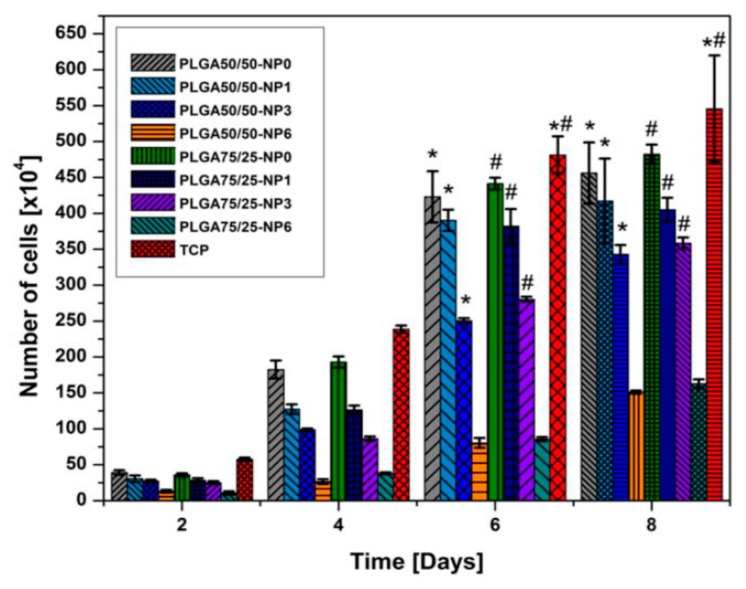
Proliferation of human dermal fibroblasts (HDFs) determined by AlamarBlue test. * Significant against cell proliferation on PLGA 50/50–6%AgNPs scaffold at *p* ≤ 0.05; # Significant against cell proliferation on PLGA75/25–6%AgNPs scaffold at *p* ≤ 0.05. NP: AgNPs. Reproduced with permission from [172], Copyright Wiley, 2015.

Almajhdi et al. electrospun PLGA/AgNPs fibrous mats containing 1 wt% and 7 wt% AgNPs [171]. They then assessed antibacterial activities of PLGA nanocomposite fibers against *E. coli*, *S. aureus*, *Bacillus cereus*, *Listeria monocytogenes* and *Salmonella typhimurium* using the disk diffusion technique. They reported that PLGA/7 wt% AgNPs mat exhibited inhibitory effect on all bacterial strains. Gora et al. incorporated 1, 3 and 6 wt% AgNPs into PLGA 50/50 and PLGA 75/25 fibrous scaffolds prepared by electrospinning [172]. PLGA 50/50 exhibited a much faster degradation rate than PLGA 75/25 due to its higher PGA content. The bactericidal activity of the fibrous mats was evaluated against *P. aeruginosa*, *K. pneumoniae*, *S. saprophyticus*, and *E. coli*. Both the PLGA nanocomposite scaffolds with 1 wt% AgNPs exhibited no antibacterial activity against *S. saprophyticus*, *E. coli*, and *P. aeruginosa*. Scaffolds contained AgNPs contents ≥ 3 wt% showed antibacterial properties for some strains when compared to neat PLGA 50/50 and PLGA 75/25 copolymers. Figure 19 showed the diffusion disk results against *P. aeruginosa* for PLGA 50/50 and PLGA 75/25 nanocomposite mats containing different AgNPs loadings. Apparently, the nanocomposite scaffolds with 6 wt% AgNPs were found to inhibit the bacterial growth effectively. However, nanocomposite scaffolds with 6 wt% AgNPs were more toxic to human dermal fibroblasts (HDFs) when compared with those filled with 1 wt% and 3 wt% of AgNPs (Figure 20). 

**Figure 21:**
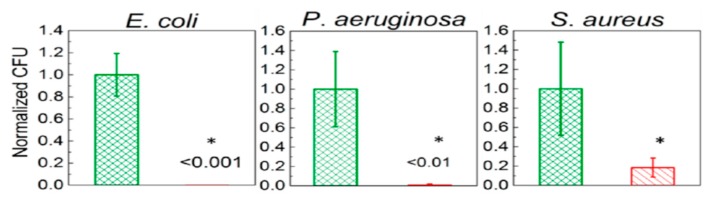

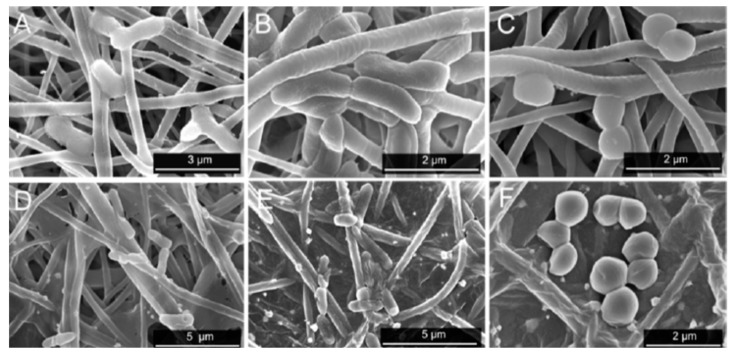
(**Top Row**) Number of attached live cells on PLGA-chitosan (green column) and PLGA-chitosan/GO-AgNPs (red column) mats upon exposure to *E. coli*, *P. aeruginosa*, and *S. aureus* bacteria. (**Bottom Row**) SEM images of *E. coli* (**A**,**D**), *P. aeruginosa* (**B**,**E**), and *S. aureus* (**C**,**F**) attached on the PLGA-CS (**A**–**C**) and PLGA-CS/GO-AgNPs (**D**–**F**) mats. * denotes statistically significant difference between PLGA-CS/GO-AgNPs and PLGA-CS (control); *p* < 0.05. Reproduced with permission from [173], Copyright American Chemical Society, 2015.

**Figure 22:**
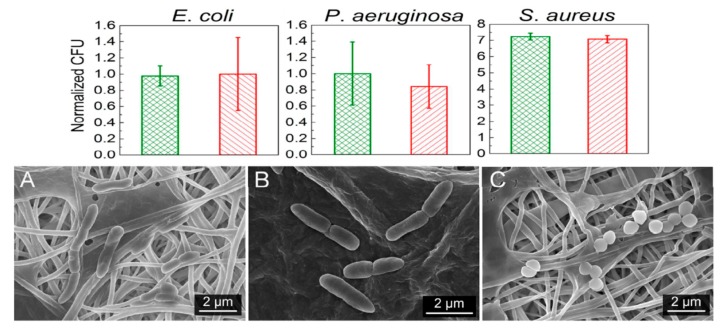
(**Top Row**) Number of attached live cells on PLGA-chitosan (green column) and PLGA-chitosan/GO (red column) mats upon exposure to *E. coli*, *P. aeruginosa*, and *S. aureus* bacteria. (**Bottom Row**) images of *E. coli* (**A**), *P. aeruginosa* (**B**), and *S. aureus* (**C**) attached on the PLGA-CS/GO samples. Reproduced with permission from [173], Copyright American Chemical Society, 2015.

Recently, de Faria et al. electrospun PLGA/chitosan (CS) fibers of 356 nm diameter. The fibrous mat was subsequently functionalized with GO-AgNPs via a chemical reaction between the carboxyl groups of GO and the primary amine functional groups on the PLGA−CS fibers using 3-(dimethylamino) propyl-*N*′-ethylcarbodiimide hydrochloride and *N*-hydroxysuccinimide as cross-linking agents [173]. Figure 21 shows the bactericidal activity of PLGA-CS/GO-AgNPs and PLGA−CS (control) mats treated with *E. coli*, *P. aeruginosa*, and *S. aureus* strains for 3 h. The PLGA-CS/GO-AgNPs mat inactivates Gram-negative *E. coli* and *P. aeruginosa* over 98%, but with a lower bactericidal rate of 79.4 ± 6.1% against Gram-positive *S. aureus*. This is due to the difference in cell wall structures of bacterial strains. Comparing with intact bacteria on PLGA−CS fibers (Figure 21A–C), SEM images reveal that gram-negatives adhered on PLGA-CS/GO-AgNPs fibers disintegrate and lose their structural integrity (Figure 21D–F). The typical rod-shaped structure of Gram-negatives is destroyed, leaving only a small portion of cell debris. In contrast, few bacterial debris are seen in the SEM image of *S. aureus* (Figure 21F). Figure 22 shows the bactericidal activity of PLGA-CS/GO and PLGA−CS (control) mats treated with *E. coli*, *P. aeruginosa*, and *S. aureus* strains for 3 h. The bacteria populations remain almost unchanged by treating with PLGA-CS/GO mat. Furthermore, SEM micrographs reveal that all bacterial cells adhered on the PLGA-CS/GO samples remain intact (Figure 22A–C). There exists no membrane damage or a loss of structural integrity for the bacteria attached on these fibers. Hence, the GO sheets are ineffective to kill all bacteria strains studied. This is due to the GO sheets are covalently linked to the PLGA-CS fibers through a stable amide bond during the nanocomposite preparation [172]. Such a covalent amide bond restricts the mobility of GO sheets, thereby reducing their bactericidal effect through direct contact mechanisms. 

### 3.3. PCL-Based Nanocomposites

Hydrophobic PCL is one of biodegradable aliphatic polyesters with a great research interest due to its ease of processability, excellent blend compatibility and low cost [174]. In this respect, PCL is commonly employed as a material for fabricating wound dressings, scaffolds and drug delivery carriers using extrusion, electrospinning and additive manufacturing techniques [174,175,176]. However, PCL exhibits very slow hydrolysis rate and takes 2 to 4 years for its complete degradation [175]. By adding GO with abundant oxygen functional groups, the degradation rate can be reduced substantially. The oxygenated groups of GO promote the scission of polymer chains, thereby resulting in a faster hydrolytic degradation of PCL [177]. 

#### 3.3.1. PCL/AgNPs Fibrous Mats

Augustine et al. studied antibacterial behavior of electrospun PCL/AgNPs mats containing 0.05–1 wt% AgNPs using disk diffusion method [178]. The PCL/1 wt% AgNPs mat exhibited excellent bactericidal activity against both *S. aureus* and *E. coli*. Dobrzanski et al. also reported the effectiveness of AgNPs fillers of electrospun PCL/AgNPs mats in killing *S. aureus* and *E. coli* [179]. More recently, Binkley et al. electrospun PCL/AgNO_3_ fibrous mats followed by air plasma treatment to reduce silver ions to AgNPs [180]. The fabrication process involved direct incorporation of AgNO_3_ solution into PCL dispersion during electrospinning, and AgNO_3_ was converted to AgNPs using air plasma treatment of as-spun fibers. The PCL/AgNO_3_ mat treated with air plasma for 5 min was found to inhibit the growth of *S. pneumoniae* as a result of plasma nucleated AgNPs within the fibers [180]. 

Electrospun fibers are versatile dressing materials with multiple functionality for wound treatment. The wound dressings should provide a suitable moisturized environment for healing by absorbing excess exudates, and killing bacteria. Very recently, Du et al. employed an electrospinning setup with two spinnerets to form PCL/polyvinyl alcohol (PVA) fibrous mats with a mixture of fibers, and loaded with AgNPs for mice wound healing [181]. Hydrolytic PVA maintains a moist wound environment to promote fibroblast adhesion, while PCL provides structural stability to impart mechanical strength to the resulting mats. Neat PCL/PVA fibrous mat is ineffective in inhibiting *E. coli* and *S. aureus* growth as expected. Hybrid PCL/PVA-AgNPs mat exhibits good bactericidal activity against *E. coli* and *S. aureus*. MTT assay using murine fibroblasts (NIH-3T3) revealed that the cell viability reduces considerably in hybrid PCL/PVA-AgNPs mat compared to neat PCL/PVA. Furthermore, the wound treated with hybrid PCL/PVA-AgNPs mat heals faster than that with neat PCL/PLA on the basis of in vivo wound healing test using a rat model. 

**Figure 23:**
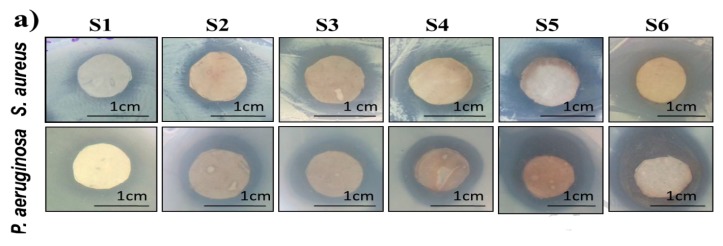

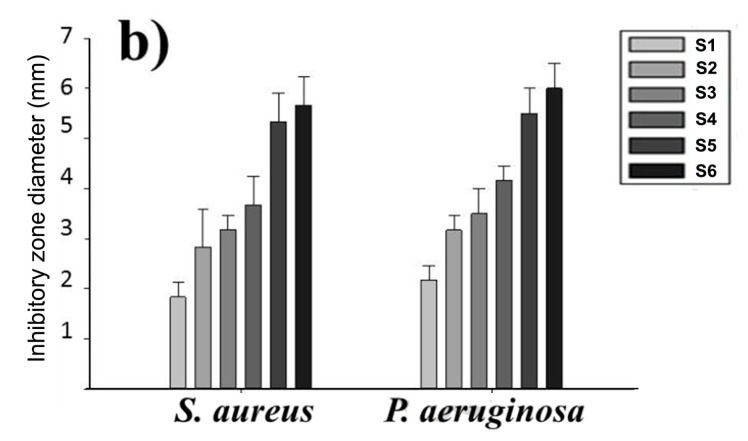
Images of S1, S2, S3, S4, S5 and S6 coated PCL mats showing (**a**) inhibition zones (**b**) zone diameters against *S. aureus* and *P. aeruginosa*. Reproduced with permission from [149]. Copyright Elsevier, 2018.

**Figure 24:**
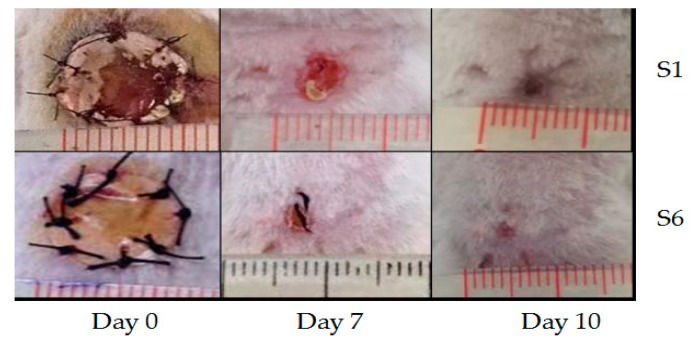
Photographs showing the contraction of dorsal wounds treated with S1 mat (**top panel**), and S6 mat (**bottom panel**) at day 0, day 7 and day 10, respectively. Reproduced with permission from [149]. Copyright Elsevier, 2018.

AgNPs can also be deposited on electrospun PCL fibers by means of coating process [149,182,183]. Nhi et al. electrospun PCL nanofibers followed by air plasma treatment. Plasma treated PCL fibers were immersed in the gelatin (Gel)-AgNPs solution for 3–50 min to obtain an optimal immersion time; the resulting sample was labeled as S1 [149]. The immersion processes were repeated for 2 to 6 cycles to achieve multiple coating layers of Gel-AgNPs on the PCL fibers. The PCL mats prepared from successive immersion in the Gel-AgNPs solution for 2 to 6 cycles were denoted as S2, S3, S4, S5 and S6, respectively. The antibacterial activity of these coated samples against *S. aureus* and *P. aeruginosa* was examined using agar diffusion method. Figure 23a showed the photographs of agar plates displaying bacterial inhibition zones of S1, S2, S3, S4, S5 and S6 samples against both bacterial strains. The diameters of inhibition zones of those samples were depicted in Figure 23b. Apparently, S6 sample exhibited maximum inhibited zone diameters of 5.8 mm and 5.6 mm upon contacting with *P. aeruginosa* and *S. aureus*, respectively. Thus, antibacterial efficacy of coated PCL fibers depended greatly on the number of Gel-AgNPs layers on their surfaces. In vivo wound healing performance was also performed by creating incision wounds on the backs of mice, then covering the wounds with S1 and S6 samples. The open wound created in other mouse without coated sample was used as the control (Figure 24). As can be seen, S6 mat healed the wound significantly faster than the S1 at day 7. At day 10, the wound covered with S6 was contracted markedly. 

**Figure 25:**
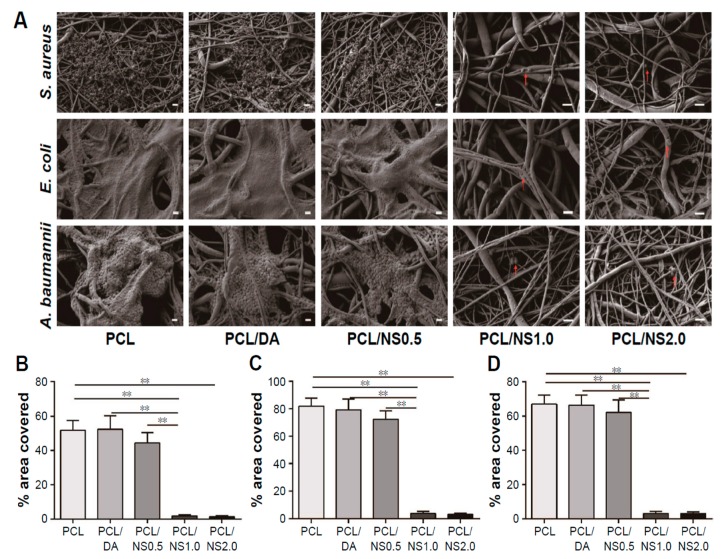
(**A**) SEM images of biofilms formed on PCL, PCL/DA, PCL/NS0.5, PCL/NS1.0 and PCL/NS2.0 mat surfaces; Scale bars: 1 μm. Red arrows indicate adherent bacteria. Measured areas covered by the biofilms of (**B**) *S. aureus*, (**C**) *E. coli* and (**D**) *A. baumannii*. ** *p* < 0.01. Reproduced with permission from [183], Dove Medical Press Limited under the Creative Commons Attribution—Non-Commercial (unported, v3.0) License.

More recently, Liu et al. electrospun PCL fibrous mats, immersed them in a dopamine (DA) solution followed by final dipping in AgNO_3_ solutions of 0.5 mM, 1 mM and 2 mM concentrations [183]. Dopamine with its mussel adhesive behavior was used to reduce silver ions to AgNPs and then deposited them on the material surfaces [184]. The DA- and AgNO_3_-treated PCL mats of different silver salt contents were designated as PCL/DA, PCL/NS0.5, PCL/NS1.0 and PCL/NS2.0, respectively. The antibacterial activity of such coated scaffolds against *S. aureus*, *E. coli* and *Acinetobacter baumannii* was investigated. *A. baumannii* is a gram-negative bacterium that induces infections typically in intensive care units and healthcare settings for patients with weakened immune system. The nosocomial infections cause pneumonia, bacteremia, and skin diseases [185]. Figure 25A shows the effects of PCL, DA- and AgNPs-coated PCL mats upon exposure to three bacterial strains. Biofilm formation due to extensive bacterial colonization of those strains can be readily seen on the surfaces of PCL, PCL/DA and PCL/NS0.5 mats, especially gram-negative bacteria. In contrast, PCL/NS1.0 and PCL/NS2.0 mats are more effective to inhibit the biofilm formation. Very few bacteria as indicated by red arrows are found to attach on their surfaces. Furthermore, the areas covered by biofilms on the PCL/NS1.0 and PCL/NS2.0 mat surfaces are markedly smaller than those on the PCL, PCL/DA and PCL/NS0.5 surfaces (Figure 25B). The bacterial inhibitory effect of PCL mats is ascribed to the released Ag^+^ ions from coated AgNPs. However, PCL/NS2.0 mat can induce cytotoxicity in murine fibroblasts on the basis of Cell Counting Kit-8 (CCK8) assay results. This is attributed to a higher amount of Ag^+^ ions released from the PCL/NS2.0 fibrous mat over that from the PCL/NS0.5 and PCL/NS1.0 mats. 

**Figure 26:**
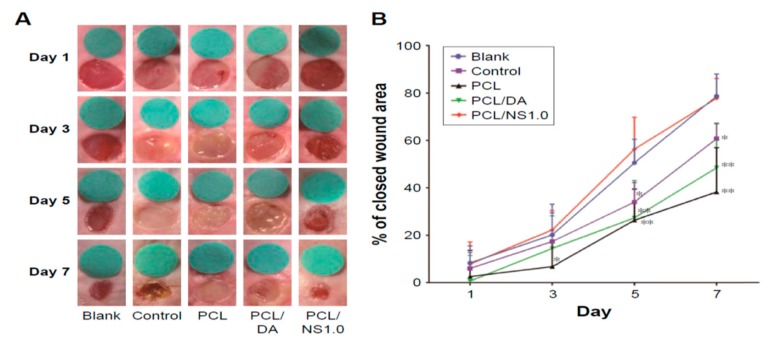
(**A**) Representative macroscopic images of mice wounds from the blank, control, PCL, PCL/DA and PCL/NS1.0 groups. (**B**) In vivo quantitative wound closure in mice at different time points. Data are presented as mean ± SD (*n* = 5). Significant differences exist between PCL/NS1.0 and the control, as well as PCL and PCL/DA groups, * *p* < 0.05, ** *p* < 0.01.

Liu et al. also carried out in vivo wound healing assessment in BALB/c adult mice through the incision of two 6 mm-diameter full-thickness wounds on either side of the back of each mouse. 10 µL of *E. coli* or *S. aureus* of 10^8^ CFU/mL was dropped respectively to each wound. Thereafter, the wounds were covered with sterile PCL, PCL/DA or PCL/NS1.0 mats. The mice without bacterial suspension and wound dressing treatment were labeled as the blank group, while those treated only with bacterial suspension were designated as the control group. Figure 26A showed that PCL/NS1.0 mat was more effective in healing the wounds than PC/DA and PCL mats at day 7. The wound contraction was quantified, and the wound closure area revealed that PCL/NS1.0 mat promoted fast healing than other mat samples due to the durable release of Ag^+^ ions for killing bacteria (Figure 26B). 

**Figure 27:**
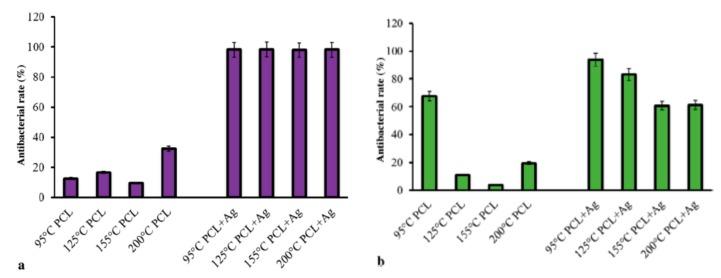
Antibacterial rates of PCL and AgNPs-loaded PCL fibrous mats prepared by pressurized melt gyration against (**a**) *E. coli* and (**b**) *P. aeruginosa* after 2 h of exposure. Reproduced with permission from [39]. Copyright Wiley-VCH, 2016.

Xu at al. employed pressurized melt gyration process to prepare PCL and AgNPs (0.01 vol.%)-loaded PCL fibrous mats at four different temperatures (95, 125, 155, and 200 °C) under two rotating speeds (24,000 and 36,000 rpm) and working pressures (0.01 and 0.02 MPa) [39]. They then determined antibacterial activity of those fibrous scaffolds against *E. coli* and *P. aeruginosa* (Figure 27a,b). PCL fibrous mats prepared at different temperatures had low antibacterial activity against *E. coli* as expected. However, all AgNPs-loaded PCL mats exhibited nearly 100% antibacterial rate against *E. coli*. The antibacterial activity of AgNPs-loaded PCL mats gradually reduced with an increase in spinning temperature for *P. aeruginosa*. The antibacterial activity of AgNPs-loaded PCL mats prepared at different temperatures was reported to diminish after 24 h for both microorganisms due to their low AgNPs content [39]. 

#### 3.2.2. PCL/RGO-AgNPs Nanocomposites

As aforementioned, GO sheets with a lateral dimension of several micrometers act as effective sites for nucleating AgNPs and for preventing AgNPs from agglomeration. The uniform dispersion of AgNPs on the GO sheets leads to higher antibacterial efficacy when compared with AgNPs or GO alone [138]. In this respect, GO–AgNPs appears as a new generation of bactericidal nanomaterial over AgNPs. Similarly, conducting RGO-AgNPs sheets show good bactericidal and electrical properties due to the synergetic effects of silver and graphene [186]. Very recently, Kumar et al. designed antibacterial wound dressings based on PCL/RGO-AgNPs by mixing RGO–AgNPs dispersion with the PCL solution, followed by solvent casting and compression molding. The weight fraction of RGO and AgNPs varied from 1% to 5% of PCL polymer [187]. Thus PCL/RGO_1, PCL/RGO_3 and PCL/RGO_5 contained 1 wt%, 3 wt% and 5 wt% RGO, while PCL/Ag_1, PCL/Ag_3 and PCL/Ag_5 had 1 wt%, 3 wt% and 5 wt% AgNPs, respectively. In addition, PCL/RGO_Ag_1, PCL/RGO_Ag_3 and PCL/RGO_Ag_5 contained 1 wt% RGO and 0.23 wt% AgNPs, 1 wt% RGO and 0.69 wt% AgNPs, as well as 1 wt% RGO and 1.15 wt% AgNPs, respectively. 

**Figure 28:**
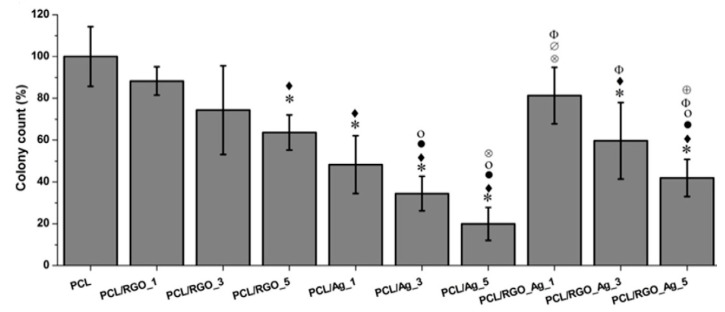
Colony count on composite surfaces incubated with *E. coli* for 3 h with respect to neat PCL. Statistically significant differences (*p* < 0.05) compared to PCL, PCL/RGO_1, PCL/RGO_3, PCL/RGO_5, PCL/Ag_1 and PCL/Ag_3 and PCL/Ag_5, PCL/RGO_Ag_1 are indicated by *, ♦, •, ο, ⊗, ∅, Φ and ⊕, respectively. Reproduced with permission from [187], Copyright Elsevier, 2016.

**Figure 29:**
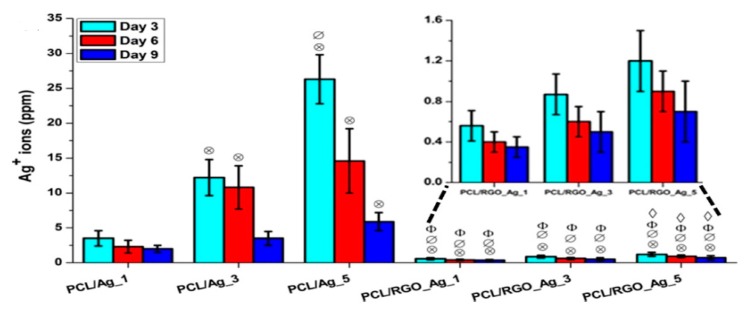
Released silver ions from PCL/Ag and PCL/RGO-Ag composite systems in pure water at 37 °C. Inset shows the enlarged view for values for PCL/ RGO-Ag system. Statistically significant differences (*p* < 0.05) compared to PCL/Ag_1 and PCL/Ag_3 and PCL/Ag_5 and PCL/RGO_Ag_1 are indicated by ⊗, ∅, Φ and ♢, respectively. Reproduced with permission from [187], Copyright Elsevier, 2016.

The antibacterial activity of all compression molded nanocomposites films against *E. coli* was shown in Figure 28. The addition of 5 wt% RGO led to a 37% decrease in bacterial population with respect to neat PCL. The PCL/AgNPs system had the largest reduction in bacterial count. For instance, PCL/Ag_5 and PCL/Ag_3 samples exhibited a decrease of 81% and 70% with respect to PCL. The hybrid PCL/RGO-Ag system also exhibited adequate bactericidal activity, e.g., PCL/RGO_Ag_5 film with a 59% reduction in colony count over PCL. The bactericidal effect of both the PCL/Ag and hybrid PCL/RGO-Ag systems arose from the Ag^+^ ions released from AgNPs fillers of both composite systems (Figure 29). The amount of released Ag^+^ ions from the PCL/AgNPs system increased with increasing filler loadings, but decreased with increasing immersion time. The PCL/Ag_3 and PCL/Ag_5 samples contained 3 wt% AgNPs and 5 wt% AgNPs, respectively, serving abundant silver sources for releasing Ag^+^ cations. The hybrid PCL/RGO-AgNPs system with a uniform dispersion of RGO in the PCL matrix had a much slower ion released rate compared with the PCL/AgNPs system. For instance, PCL/RGO_Ag_5 film contained 1 wt% RGO and 1.15 wt% AgNPs released lower Ag^+^ ions concentration than the PCL/Ag_1. The RGO filler with a deficiency in oxygenated groups imparted hydrophobicity to PCL, leading to an increase in water contact angles of resulting PCL/RGO composites. Thus RGO additions could affect the wettability of aliphatic polyesters. As aforementioned, GO with abundant oxygenated groups rendered PLA/GO less hydrophobic. Moreover, the incorporation of AgNPs to the PLA/GO system further reduced the water contact angle and enhanced Ag^+^ ion concentration as shown in Figure 12a,b. This facilitated the release of Ag^+^ ions so that the ion concentration increased with increasing immersion time [165]. 

**Figure 30:**
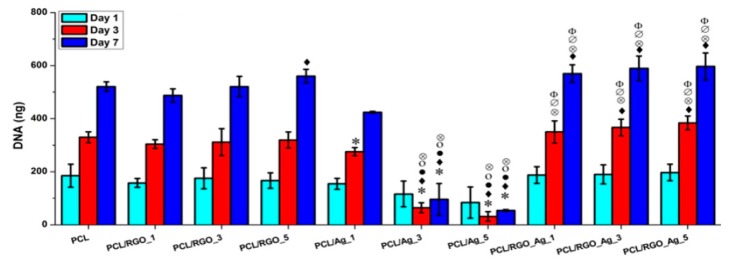
DNA quantification of hMSCs cultured on PCL and its composites for 1, 3 and 7 days. Statistically significant differences (*p* < 0.05) compared to PCL, PCL/RGO_1, PCL/RGO_3, PCL/RGO_5, PCL/Ag_1 and PCL/Ag_3 and PCL/Ag_5 are indicated by *, ♦, •, ο, ⊗, ∅ and Φ, respectively. Reproduced with permission from [187], Copyright Elsevier, 2016.

The amount of released Ag^+^ ion ions from the PCL/Ag system affects the biocompatibility and induces cytotoxicity in stem cells. Figure 30 shows the DNA content of primary bone marrow-derived human mesenchymal stem cells (hMSCs) cultured on compression molded PCL-based nanocomposite films. hMSCs are multipotent stem cells capable of differentiating into osteoblasts, adipocytes, or chondrocytes [188]. At day 1, DNA content of hMSCs cultured on all samples shows little variations. At day 7, DNA content of hMSCs cultivated on the PCL/Ag_3 and PCL/Ag_5 samples is greatly reduced, showing the toxicity of AgNPs to the cells. In contrast, DNA content of hMSCs on the PCL/RGO and PCL/RGO-Ag systems is the same or slightly higher than that of neat PCL. This demonstrates that RGO and hybrid RGO-Ag nanofillers exert no toxicity to hMSCs. The DNA quantification results agree reasonably with those of silver ion release measurements. Both PCL/Ag_3 and PCL/Ag_5 samples exhibit a much higher released Ag^+^ ion concentration than the PCL/RGO-Ag system. PCL/Ag_3 and PCL/Ag_5 samples release more than 12 and 26 ppm Ag^+^ ions respectively at day 3, while 11 and 15 ppm Ag^+^ ions respectively at day 6, thereby causing cytotoxicity. Recently, Pauksch et al. reported that AgNPs concentration of 10 ppm can induce toxicity to hMSCs [189]. However, Greulich et al. pointed out that released Ag^+^ ions from silver acetate with a concentration of 2.5 ppm decrease the viability of hMSCs to about 35% after 24 h cultivation [190]. Silver ions with concentrations ≤ 1.5 ppm show 100% cell viability, i.e., no cytotoxicity. In contrast, AgNPs with concentrations ≤ 30 ppm show 100% vital cells. Thus, silver ions are more toxic than AgNPs against hMSC. From Figure 29, it appears that hybrid PCL/RGO-Ag samples induce no cytotoxicity to hMSCs, because they release low Ag^+^ ions (0.4–1.2 ppm) over a period of 10 days. However, PCL/Ag_3 and PCL/Ag_5 films with 3 wt% AgNPs and 5 wt% AgNPs loadings induce cytotoxicity due to the release of large amounts of Ag^+^ ions. This raises concerns about biosafety of PCL/AgNPs films for their potential use as wound dressings in clinical sector, especially those with high filler loadings. 

## 4. Mechanical Properties

Aliphatic polyester nanocomposites for bone fixation device and bone scaffold applications should exhibit high mechanical strength and elastic modulus for supporting bone cell regeneration and growth. The tensile properties of solid aliphatic polyester films and porous scaffolds can be enhanced by adding low loadings of GOs and AgNPs [165,191,192,193,194]. Li et al. fabricated PLA/0.5 wt% GO and PLA/0.5 wt% GO-g-PLA composites using solvent casting followed by compression molding into thin films of 1 mm thickness. Functional PLA/GO-g-PLA was prepared by polycondensation of the l-lactic acid monomer with lyophilized GO [191]. By adding 0.5 wt% GO, the tensile strength and elongation of break of PLA increase from 35 MPa to 53 MPa (an improvement of 51.4%), and from 6.50% to 8.91% (an enhancement of 37.1%), respectively. The increase in tensile strength of PLA can be attributed to the reinforcing effect of GO with a high stiffness of 207.6 ± 23.4 GPa [103]. A further increase in tensile strength and tensile ductility of PLA can be achieved by adding 0.5 wt% GO-g-PLA. Thus the PLA/0.5 wt% GO-g-PLA film exhibits a high tensile strength of 72 MPa (an increase of 105.7%) and elongation at break of 14.48% (an improvement of 122.8%). These enhancements arise from the grafted PLA chains, which improve the interaction between GO and PLA, leading to a uniform dispersion of GO-g-PLA nanofillers in the PLA matrix. As such, the fillers can bear applied load transferred from the PLA matrix during tensile testing. This behavior is widely known as the stress-transfer mechanism. The tensile properties of PLA/GO nanocomposite films are listed in Table 1. Wan and Chen also employed combined solvent casting and compression molding to prepare PCL/GO nanocomposite films [192]. The tensile parameters of PCL including modulus of elasticity, tensile strength and tensile elongation increase from 209 ± 21 MPa, 14.2 ± 1.6 MPa and 554 ± 72%, to 311 ± 25 MPa, 25.5 ± 3.2 MPa and 732 ± 61%, respectively due to the addition of 0.5 wt% GO. A similar reinforcing effect can be obtained by adding 1 wt% and 2 wt% GO to PCL. The reinforcing effect of GO on PCL results from the establishment of hydrogen bonding between the GO and PCL on the basis of Fourier transform infrared spectroscopic results, leading to a uniform dispersion of GO fillers in the PCL matrix. Li et al. prepared neat PCL and PCL/GO nanocomposites using in situ polymerization process, followed by hot pressing to produce dense films. They demonstrated that the incorporation of 0.5 wt% GO into PCL imparts the resulting nanocomposite with higher modulus of elasticity and tensile strength over neat PCL. The elastic modulus of PCL increases from 414 ± 24 MPa to 496 ± 22 MPa, while the tensile strength increases from 13.9 ± 1.2 MPa to 20.7 ± 2.6 MPa by adding 0.5 wt% GO [194]. In this respect, the stiffness and tensile strength of PCL are enhanced by 20% and 50%, respectively. By increasing GO loadings to 1 wt% and 2 wt%, the tensile strength and elastic modulus of PCL are further improved as expected. Table 1 summarizes the tensile properties of representative PLA/GO and PCL/GO nanocomposite films. The tensile properties of PLA/0.5 wt% AgNPs are included for the purpose of comparison. 

For electrospun polyester nanocomposite scaffolds, the presence of high volume fraction of fined pores leads to a drastic reduction in their tensile strength and elastic modulus. Tjong and coworkers investigated the tensile properties of electrospun PLA scaffolds reinforced with AgNPs, GO and GO-AgNPs fillers [165]. The tensile properties of those fibrous mats are tabulated in Table 2. Solid PLA typically exhibits elastic modulus of 3.1 GPa and tensile strength of 47.8 MPa [193]. In contrast, electrospun PLA mat shows a significant lower elastic modulus and tensile strength of 8.70 MPa and 0.76 MPa, respectively. However, both the AgNPs and GO fillers are beneficial in enhancing the stiffness and strength of PLA fibrous mats. For the PLA/AgNPs system, the tensile strength and stiffness increase with increasing AgNPs loadings. The AgNPs fillers confine the segmental movement of the molecular chains of PLA during tensile testing, leading to enhanced tensile strength of the nanocomposites. Comparing with the PLA/AgNPs system, PLA/1 wt% GO-AgNPs hybrid nanocomposites exhibit even higher tensile performance. The PLA/1 wt%GO-7 wt% AgNPs hybrid shows the highest modulus of elasticity and tensile strength of 1.21 GPa and 5.46 MPa, respectively. Similarly, the tensile properties of electrospun PCL mats can also be improved by adding GO or AgNPs [178,192,195]. It can be concluded that the AgNPs, GO and GO-AgNPs nanofillers increase the tensile strength and elastic modulus of polyester nanocomposites either in the form of dense films or porous fibrous mats. Enhanced mechanical strength of aliphatic polyester nanocomposites is necessary to support bone growth and new bone generation, and to offset the weakening of nanocomposites due to the degradation of polyester matrix in human body. 

## 5. Future Challenges

Development of biodegradable polymer nanocomposites against antibiotic-resistant bacteria is a main focus of interest in bone tissue engineering. Significant advances have been made recently in the development and processing of antibacterial aliphatic polyester nanocomposites with AgNPs, GO and GO-AgNPs fillers for fabricating internal fixation devices, scaffolds and wound dressings. The translation of those antibacterial nanocomposites to the clinic still face many challenges. However, despite the potential benefits of AgNPs with bactericidal activity, there is a major concern regarding their cytotoxicity to human tissues. As mentioned, injection molded PLA/18 wt% nHA–(2–25 wt%)AgNPs nanocomposites exhibit strong cytotoxic effects on osteoblasts when the AgNPs filler loadings ≥ 18 wt%. No cytotoxicity is observed at AgNPs filler contents ≤ 10 wt% (Figure 9a) [154]. Solvent-cast PLGA/7 wt% AgNPs nanocomposite exhibits cytotoxicity to fibroblasts (L929) and osteoblasts (Saos-2) cells [170]. Compression-molded PCL/AgNPs with 3 wt% and 6 wt% AgNPs show cytotoxicity to hMSCs (Figure 28). The cytotoxicity of AgNPs in the PCL/Ag system can be minimized by simultaneously adding RGO, forming hybridized RGO-AgNPs fillers [187]. 

Alternatively, AgNPs can be replaced by other metal nanoparticles such as copper nanoparticles to reduce toxic effects on osteoblasts and stem cells. Copper is an essential trace element for human body that contains about 100 mg. However, copper is toxic at high dose levels [196]. Comparing with expensive silver, it is more cost effective to employ cheaper copper for eliminating bacteria. Copper and its alloys have been used for centuries as antimicrobial materials. Copper alloys have been identified by U.S. Environmental Protection Agency (EPA) as antimicrobial materials in the healthcare environment of hospitals [197]. Copper alloys can reduce bacterial infections in hospital rooms, so they are widely employed as hospital touch surface materials such as bathroom fixtures, bed rails, door handles, drug trolleys, etc. [198]. The accumulation of large amounts of copper ions is responsible for contact killing of bacterial cells attached to the copper surfaces including MRSA [199,200]. 

Copper nanoparticles (CuNPs) have been found to exhibit antibacterial effect against several bacteria strains and microorganisms [201,202,203]. Yoon et al. studied bactericidal behavior of AgNPs and CuNPs against Gram-negative *E. coli* and Gram-positive *Bacillus subtilis* [204]. They reported that CuNPs (100 nm) exhibit higher antibacterial activity than AgNPs (40 nm). Cioffi et al. carried out a preliminary study on biocidal behavior of several polymer/CuNPs composites with non-degradable matrices based on poly(vinylmethyl ketone), poly(vinylidene fluoride) and poly(vinyl chloride) [205]. The bactericidal behavior of these composites against *E. coli* and *S. aureus* depends largely on the CuNPs loadings and an effective release of copper ions from the CuNPs. Cai et al. found that copper ions released from CuNPs fillers embedded in non-degradable, low-density polyethylene are responsible for antibacterial activity [206]. For biodegradable natural chitosan with abundant amino groups, it can serve as a capping agent and anchoring site for CuNPs, because amino moieties favor the formation of stable chelate complexes with copper ions [207]. 

CuNPs are highly unstable in solutions and oxidize easily under atmospheric conditions to yield copper oxide on their surfaces. In general, CuNPs are protected by stabilizing or capping agents such as organic ligands, surfactants, or polymers that can form complexes with copper ions during the synthesis. The capping agents bind onto the surfaces of CuNPs, forming a well coated particles. Surfactants such as sodium dodecyl sulfate, cetyltrimethylammonium bromide, polyethylene glycols and polyvinylpyrrolidone are employed to stabilize CuNPs [208,209]. This gives rise to an issue relating the solubility of surfactant-capped CuNPs in organic solvents used to prepare aliphatic polyester/CuNPs nanocomposites and electrospun scaffolds. The selection of suitable surfactant type and its concentration is crucial to disperse CuNPs in a solution for fabricating aliphatic polyester/CuNPs nanocomposites. Future works are needed to elucidate this issue. 

From the literature, extensive research studies have been made on the preparation and antibacterial characterization of electrospun aliphatic polyester nanocomposite scaffolds containing AgNPs, GO and GO-AgNPs fillers [149,163,165,170,171,181,182,183]. The main limitations of electrospun polyester nanocomposite scaffolds for bone tissue engineering applications are their small pore size and limited membrane thickness. The small pores prevent infiltration of osteoblasts into the scaffolds for bone ingrowth and vascularization [210]. Recent advances in nanotechnology enable the development of additive manufacturing process for fabricating three-dimensional aliphatic polyester scaffolds with large pores for tissue engineering applications [176,211,212]. However, no information is available in the literature regarding additive manufacturing and bactericidal properties of aliphatic polyester nanocomposite with AgNPs, GO and GO-AgNPs fillers. In this context, the development of three-dimensional aliphatic polyester nanocomposite scaffolds with antibacterial properties remains a big challenge for chemists and materials scientists. 

## 6. Conclusions

The development of biodegradable polymer nanocomposites with antibacterial properties is of crucial importance in clinical sector. This review is focused on more recent works concerning the incorporation of antimicrobial AgNPs and/or GO nanofillers into biodegradable aliphatic polyesters such as PLA, PLGA and PCL. Those antibacterial nanocomposites show potential applications for the fixation devices, scaffolds and wound dressings in bone tissue engineering. For clinical applications, dense aliphatic polyester nanocomposites with antibacterial activity for bone fixation devices can be prepared by means of solvent casting/compression molding, and melt-mixing/injection molding. Porous scaffolds are fabricated using solvent casting/porogen, electrospinning and pressurized gyration techniques. 

Device-associated infections may cause significant morbidity and mortality in patients. Infections caused by antibiotic-resistant bacteria are difficult to treat globally. By incorporating 2% AgNPs into BMP-2 coupled PLGA, the resulting graft shows efficient killing activity against vancomycin-resistant *S. aureus* [144]. Electrospun PLGA/3 wt% AgNPs mat shows sufficient antibacterial activity against *P. aeruginosa*, being the source of nosocomial pneumonia and surgical site infections [172]. Moreover, electrospun PLGA-CS/GO-AgNPs hybrid fibers can lead to a loss of membrane integrity of *P. aeruginosa* and *E. coli* [173]. AgNPs-loaded PCL mats prepared by pressurized melt gyration process has been found to exhibit nearly 100% antibacterial rate against *E. coli* [39]. In this respect, the incorporation of AgNPs and GO-AgNPs fillers into aliphatic polyesters can reduce bacterial infections of medical devices and bone scaffolds, thus decreasing overuse of antibiotics in the clinical sector. 

Finally, AgNPs and GO-AgNPs fillers of aliphatic nanocomposites act a double-edged sword having bactericidal activity, and cytotoxic effect to human cells, especially at high loading levels. Those fillers can induce cytotoxicity on fibroblasts, osteoblasts and stem cells. Alternatively, CuNPs can be used as nanofillers for aliphatic polyesters in forming antimicrobial nanocomposites. Till to present, antibacterial activity and biocompatibility of aliphatic polyester-based nanocomposites containing CuNPs remain unexplored. 

## Figures and Tables

**Table 1:** Tensile properties of PLA/GO and PCL/GO solid thin films.

Specimen	Processing Techniques	Modulus of Elasticity, MPa	Tensile Strength, MPa	Elongation, %	Ref.
PLA	Solvent casting	3118.8	47.8	5.35	[193]
PLA	Solvent casting & compression	NA	35	6.50	[191]
PLA/0.5 wt% GO	Solvent casting & compression	NA	53	8.91	[191]
PCL	Solvent casting & compression	209 ± 21	14.2 ± 1.6	554 ± 72	[192]
PCL/1 wt% GO	Solvent casting & compression	305 ± 35	21.8 ± 5.6	597 ± 45	[192]
PCL/2 wt% GO	Solvent casting & compression	442 ± 35	27.5 ± 5.7	548 ± 81	[192]
PCL	Polymerization & hot pressing	414 ± 24	13.9 ± 1.2	478 ± 42	[194]
PCL/1 wt% GO	Polymerization & hot pressing	578 ± 21	26.5 ± 2.4	402 ± 4	[194]
PLA/0.5 wt% AgNPs	Solvent casting	2811.8	44.3	6.68	[193]

NA: Not available.

**Table 2:** Tensile properties of electrospun PLA- and PCL-based fibrous scaffolds reinforced with AgNPs and/or GO nanofillers.

Specimen	Modulus of Elasticity, MPa	Tensile Strength, MPa	Elongation, %	Reference
(I) PLA-based system				
PLA	8.70 ± 0.90	0.76 ± 0.04	NA	[165]
PLA/1 wt% AgNPs	269.59 ± 6.51	1.67 ± 0.12	NA	[165]
PLA/3 wt% AgNPs	412.23 ± 8.54	3.06 ± 0.54	NA	[165]
PLA/7 wt% AgNPs	968.05 ± 12.71	4.65 ± 0.76	NA	[165]
PLA/1 wt% GO	147.78 ± 3.46	1.22 ± 0.12	NA	[165]
PLA/1 wt% GO-1 wt%AgNPs	377.09 ± 5.33	2.06 ± 0.20	NA	[165]
PLA/1 wt% GO-3 wt%AgNPs	755.40 ± 8.80	3.71 ± 0.48	NA	[165]
PLA/1 wt% GO-7 wt%AgNPs	1211.05 ± 13.53	5.46 ± 0.81	NA	[165]
(II) PCL-based system				
PCL	2.84 ± 0.87	1.24 ± 0.21	250 ± 8	[178]
PCL/0.5 wt% AgNPs	4.28 ± 1.28	2.78 ± 0.18	427 ± 16	[178]
PCL/1 wt% AgNPs	2.97 ± 0.59	1.23 ± 0.08	251 ± 11	[178]
PCL	10.5 ± 0.92	2.37 ± 0.09	NA	[192]
PCL/0.3 wt% GO	17.4 ± 1.25	4.61 ± 0.15	NA	[192]

NA: Not available.
